# Real-time high dynamic range laser scanning microscopy

**DOI:** 10.1038/ncomms11077

**Published:** 2016-04-01

**Authors:** C. Vinegoni, C. Leon Swisher, P. Fumene Feruglio, R. J. Giedt, D. L. Rousso, S. Stapleton, R. Weissleder

**Affiliations:** 1 Center for Systems Biology, Massachusetts General Hospital and Harvard Medical School, Richard B. Simches Research Center, 185 Cambridge Street, Boston, Massachusetts 02114, USA; 2 Department of Neurological, Biomedical and Movement Sciences, University of Verona, Strada Le Grazie 8, 37134 Verona, Italy; 3 Center for Brain Science, Department of Molecular and Cell Biology, Harvard University, 52 Oxford Street, Cambridge, Massachusetts 02138, USA

## Abstract

In conventional confocal/multiphoton fluorescence microscopy, images are typically acquired under ideal settings and after extensive optimization of parameters for a given structure or feature, often resulting in information loss from other image attributes. To overcome the problem of selective data display, we developed a new method that extends the imaging dynamic range in optical microscopy and improves the signal-to-noise ratio. Here we demonstrate how real-time and sequential high dynamic range microscopy facilitates automated three-dimensional neural segmentation. We address reconstruction and segmentation performance on samples with different size, anatomy and complexity. Finally, *in vivo* real-time high dynamic range imaging is also demonstrated, making the technique particularly relevant for longitudinal imaging in the presence of physiological motion and/or for quantification of *in vivo* fast tracer kinetics during functional imaging.

## Abstract (web summary)

Confocal and multiphoton fluorescence microscopy often suffers from low dynamic range. Here the authors develop a high dynamic range, laser scanning fluorescence technique by simultaneously recording different light intensity ranges. The method can be adapted to commercial systems.

## Graphical abstract

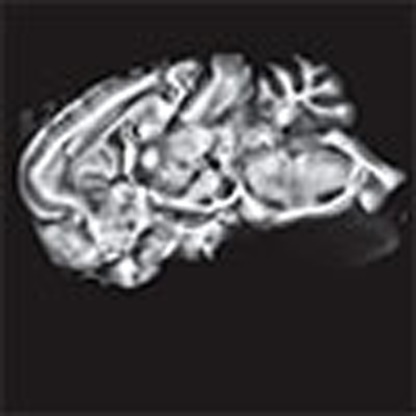

The ability to directly visualize cellular and subcellular structures and function has greatly contributed to our knowledge of biological processes^1^,^2^,^3^. Among optical imaging techniques, laser scanning fluorescence microscopy (LSM) is one of the most widely used due to its high sensitivity, resolution, and penetration depth. Two-photon microscopy in particular has enabled major advances in virtually every biological field to which it has been applied to date^4^. Most commonly, LSM techniques are optimized and acquisition parameters are chosen to display a given structure of interest. This approach works well for many applications but is disadvantageous in circumstances where structures of contrasting brightness cannot be displayed simultaneously. This is particularly true for neuronal imaging, where cell bodies are significantly larger than neuronal processes, and where there is heterogeneity in the density of cell populations resulting in high intra-scene dynamic range. Furthermore, images with low signal-to-noise ratio (SNR) will lead to the fragmentation of the neural segments. Conversely, the presence of saturated regions will result in the inability to differentiate cell bodies or processes from neighbouring cells.

Photomultiplier tubes (PMT) are ubiquitous among commercial confocal and two-photon microscopy systems, due to their low cost, high sensitivity and wide coverage of wavelengths. Therefore, a high dynamic range (HDR) imaging method that utilizes PMT technology would provide broad access to microscopists. The PMTs used in LSM have a limited detection dynamic range, typically three orders of magnitude, which determines the range of variance in the detectable fluorescence signal and thus the maximum and minimum intensities that can be simultaneously detected within a field of view^5^. For biological samples, the intra-scene dynamic range (IDR) is determined by the underlying biology and is thus dependent on the distribution and concentration of protein expression or target molecules to be imaged. Because the IDR is typically large compared with the detectable dynamic range of PMTs, images will inevitably have regions with intensities that are either saturated or below the background, leading to information loss and compromised image quality. Moreover, despite the fact that typical microscopy imaging systems provide images with 8 or 12 bits depth, the available IDR acquired from the sensor can be largely reduced by the amount of noise and background resulting in an effective dynamic range with reduced bit depth.

Avalanche photodiode detectors (APD) constitute an alternative option to PMTs, especially when operating at low photon fluxes, where PMTs suffer from a significant amount of dark noise above the shot noise limit^6^. In this regime, pulse counting detection^7^ is usually preferred, offering high SNR at low counting rates^6^. However, the dynamic range of single photon counting measurements is relatively low with a limited counting rate on the order of approximately 10^7^ counts per second^8^, confining its applications in optical microscopy to highly specialized areas where very low number of photons are present. Commercially available single photon counting instruments offer maximum count rates on the order of 10 to 100 Mega-counts per second (ref. 8) but their linearity is still limited to just 1 to 2 MHz (ref. 9). These values are insufficient to produce high-SNR images for pixel dwell times below 10 microseconds or alternatively for pixel acquisition rates higher than 100 kHz (ref. 8). Thus single photon counting is impractical for high-resolution imaging at high SNR, restricting its use to small fields of view and longer dwell times^10^. Another limiting factor is the readout rate (pixel clock rate), which gives the speed at which data can be retrieved from the detection scheme^10^. Only recently has the use of sophisticated photon counting circuity or the implementation of field programmable-gate arrays in combination with statistical processing substantially improved their dynamic range, extending photon-counting operation to higher-emission rate regimes^11^,^12^. But these methods are still early in development, far from being commercially available, and have only been applied in a few specialized studies^6^,^8^,^11^,^13^.

So far, several approaches have been developed to extend the dynamic range of optical imaging detectors, both hardware and software based. High dynamic range imaging for digital still cameras^14^,^15^, in particular, has reached the mainstream through the use of smart phones and digital single-lens reflex (DSLR) cameras, and is based on the acquisition of several images with progressively increasing exposure times (exposure bracketing). Although these techniques have found a wide range of applications, they lack the resolution and sensitivities necessary to image at the subcellular level. For fluorescence microscopy, hardware-based approaches have also been developed to extend the dynamic range of optical imaging detectors, including adaptive illumination^16^. The adaptive illumination method uses negative feedback loops in combination with analogue optical modulators to hold the average detected power at a constant level^16^,^17^. Although adaptive illumination is an elegant approach, it requires additional electronics, realignment of the setup and the presence of electro-optics modulators. Statistical approaches can also be effective at extending the linear range in photon-counting measurements during pulsed excitation^18^. Finally, a new class of recently introduced PMT tubes (H13126, Hamamatsu) appears to offer a wide dynamic range up to eight orders of magnitude.

Here, we present a new technical approach for confocal and two-photon microscopy namely, high dynamic range fluorescence laser scanning microscopy (HDR-LSM). The technique is based on the simultaneous or sequential acquisition of progressively saturated images mathematically fused into a composite HDR image. Moreover, we propose a method for simultaneous or sequential acquisition of HDR data, which requires no additional acquisition time (for the simultaneous acquisition case), and can be easily implemented on any commercially available LSM system both in two-photon and/or confocal mode. We show that HDR-LSM improves image segmentation and quantification by applying the method to neural tracing, and on samples with different sizes, anatomy and complexity. Finally, *in vivo* real-time imaging is demonstrated, allowing for longitudinal HDR imaging in the presence of physiological motion as well as for quantitative imaging of *in vivo* fast tracer kinetics.

## Results

### Imaging setup and acquisition pipeline

**Figure 1:**
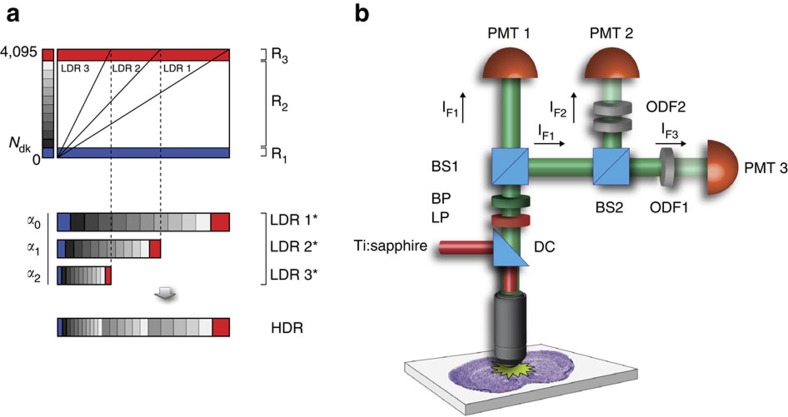
Imaging setup and image-processing principle for real-time two-photon imaging. (**a**) Principle for HDR imaging. Only a restricted portion of the detector dynamic range can be effectively used for signal quantization (*R*_2_). The dark noise (blue area, *R*_1_) limits the low signal detection, while the high intensity signal near the detector's maximum threshold is saturated (red) and is also disregarded (*R*_3_). By combining multiple images (LDR1, LDR2, LDR3) with different sensitivities (*α*_0_, *α*_1_, *α*_2_) the quantization range can be increased giving rise to a high dynamic range image (HDR). Images are simultaneously acquired from three different detectors (PMT1, PMT2, PMT3). The presence of neutral optical density filters with distinct absorptions gives rise to three images covering different areas of the sample's dynamic range. When no absorption filter is present, the limited quantization range provides high sampling resolution at low signal values while saturated regions values are instead unresolvable. Conversely, the images obtained when increased absorption is present in front of the detector, extend the range of the saturated pixels into the usable quantization interval. Meanwhile, regions within the image with low signal are instead buried into the noise. When the information from all the images is combined together, it provides an extended dynamic range and high SNR image. The dark noise level can be different for other measurements schemes. LDR* represents LDR images weighted by *α*. (**b**) Schematic representation of the two-photon imaging setup for real-time HDR imaging (Supplementary Fig. 2 and ‘Methods' section). BS, beam splitter; BP, bandpass filter; DC, dichroic mirror; LP, longpass filter; ODF, neutral optical density filter; PMT, photomultiplier tubes. Depending on the microscope setup or preferences, BS could be cube beam splitters, plate beam splitters or a combination of both. An arbitrary number of acquisition channels can be used depending on the number of PMTs available and the intra-scene dynamic range. To perform real-time HDR two-photon acquisition, a minimum number of two PMTs is required.

The acquisition and processing pipeline for HDR-LSM (Supplementary Fig. 1) consists of acquiring, simultaneously or sequentially, a series of images covering the full dynamic range of the sample (Fig. 1a), reconstructing a composite HDR image (Supplementary Note 1) for quantitative signal data analysis, and then remapping the HDR image (rHDR) for display and image feature enhancement for structural data analysis, using a global nonlinear transformation followed by a histogram equalization if further local contrast is required (Supplementary Note 2).

The imaging setup is based on a custom-modified commercial imaging system (Fig. 1b, Supplementary Figs 2–4, ‘Methods' section). Here low dynamic range images (LDR) are acquired simultaneously for the real-time acquisition scheme, or sequentially, under different detection conditions (for example, attenuation of the signal before PMT detection) such that different parts of the images progressively result in saturation (Supplementary Figs 2–4). LDR images are then corrected for the detectors' response (Supplementary Fig. 5), and combined into a composite high dynamic range image (HDR; details available in Supplementary Note 1) with a dynamic range greater than the individual LDRs. Other real-time acquisition configurations are possible (Supplementary Note 1), including the use of asymmetric non-polarizing beamsplitters (Supplementary Fig. 4). Here, the whole fluorescence signal contributes to the HDR image reconstruction being the fluorescence distributed by the beam splitters in different ratios to the PMTs.

**Figure 2:**
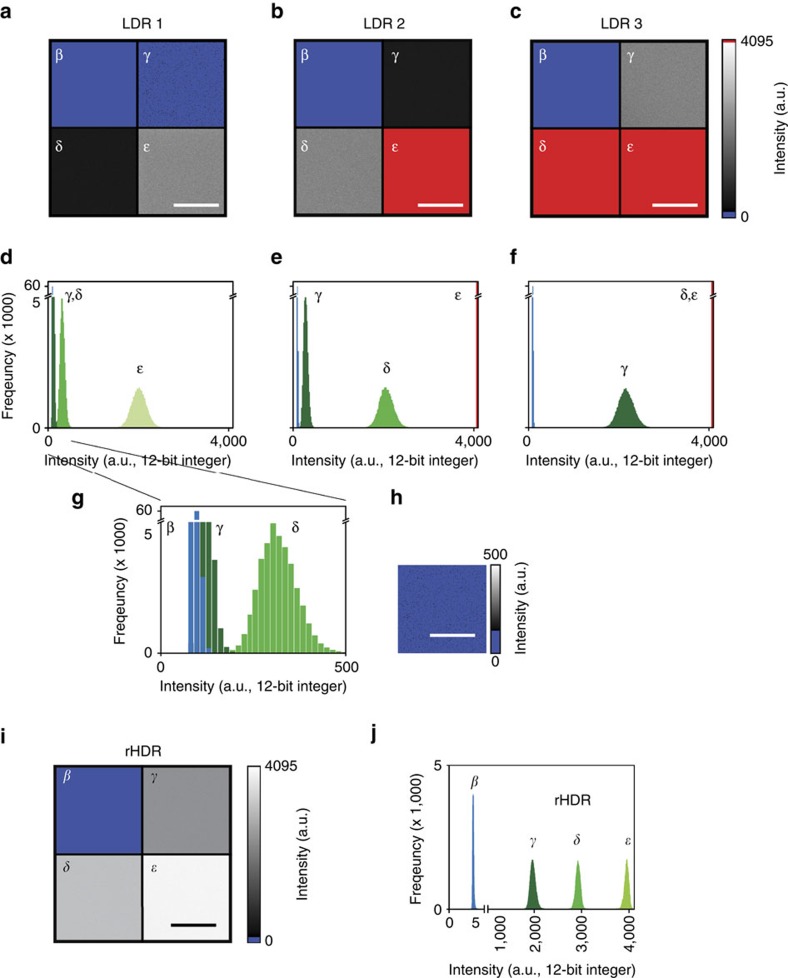
Imaging phantom. (**a**–**c**) Three low dynamic range (LDR) fluorescence images with their relative histograms (**d**–**f**), of an imaging phantom composed of four distinct non-adjacent areas with different fluorophore concentrations (Supplementary Fig. 6 and ‘Methods' section), as measured from one detector with its dynamic range accurately centred around the maximum intensity of each specific region (γ, δ, ɛ). Region γ, dark green. Region δ, green. Region ɛ, light green. (**g**,**h**) In the non-saturated image (LDR1), the signal contribution from the region γ is buried within the noise with a low SNR (re-scaled subset image of region γ is shown in **h**. (**i**,**j**) Remapped HDR image (rHDR, compressed dynamic range for visualization) obtained combining the information from the three different LDR images and displayed with a dynamic range compression mapping algorithm (‘Methods' section and Supplementary Note 2), along with its corresponding histogram. The dark noise image (region β) is the same for all phantom's mosaicked images **a**–**c**. The blue colormap threshold for the dark noise is set at the maximum of the dark noise signal. Image colour bar: blue, dark noise; red, saturation levels. Scale bar, 50 μm.

We first validated the sequential acquisition and reconstruction scheme in a phantom with regions of known fluorescence concentration (Fig. 2a–h, Supplementary Fig. 6, ‘Methods' section). In the LDR image (Fig. 2a), the signal from the region with the lowest concentration of fluorophore (γ) was buried below the background noise (β) when the highest concentration of fluorophore (ɛ) was not saturated. Conversely, when the lowest concentration was above the noise, the highest concentration was saturated (Fig. 2c). However, using HDR-LSM imaging, all three concentrations were within the observation range (Fig. 2i,j). This is accomplished by fusion of the LDR images into a composite HDR image (Supplementary Fig. 7) and then remapping the HDR image (re-mapped HDR, rHDR) for visualization (Fig. 2i)^19^,^20^,^21^,^22^,^23^,^24^. The SNR over the range of the entire image is also greatly improved and imaging is substantially faster compared with the conventional method of averaging (Supplementary Fig. 8).

### HDR two-photon imaging

**Figure 3:**
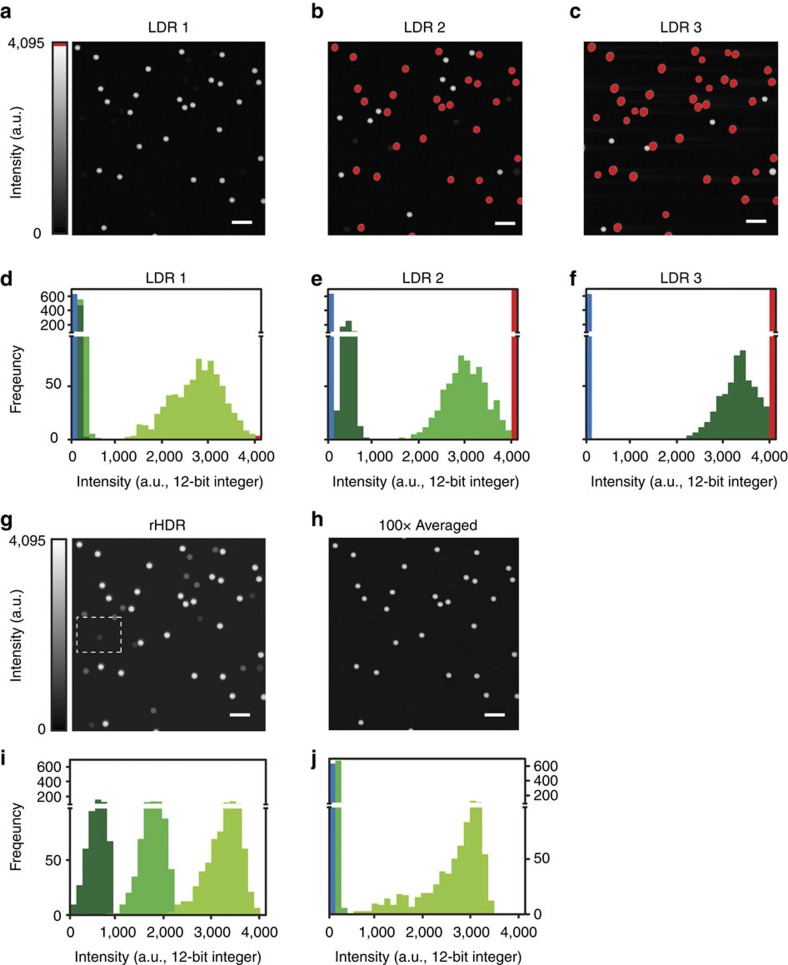
Microspheres HDR two-photon imaging. (**a**–**c**) Low dynamic range fluorescence images and (**d**–**f**) relative histograms of a mixture of three fluorescent microspheres populations with three different discrete values of fluorescence concentrations (see ‘Methods' section) centred at different intensity signals within the digitizing range. Bead population 3, light green. Bead population 2, green. Bead population 1, dark green. (**g**) Remapped HDR image (rHDR, compressed dynamic range for visualization) and (**h**) averaged (100 ×) low intensity LDR1 image. (**i**,**j**) Corresponding histograms. Image colour bar: red, saturation levels. Scale bars, 10 μm.

**Figure 4:**
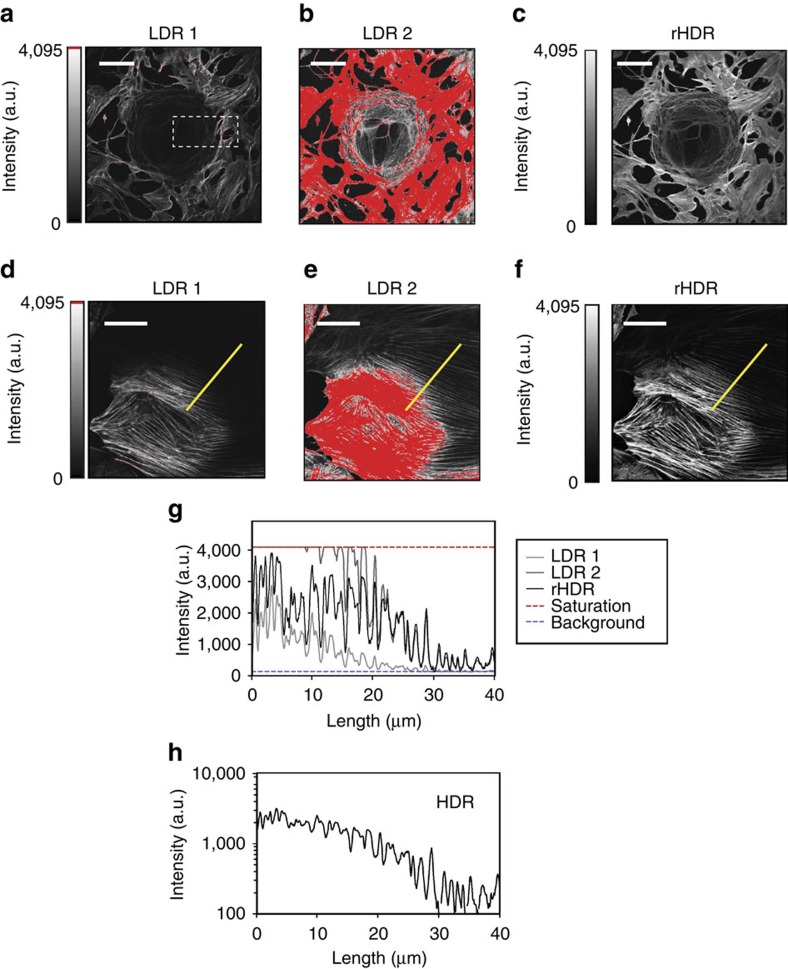
Cellular HDR two-photon and confocal imaging. (**a**–**f**) Images of BS-C-1 cells stained for actin (see ‘Methods' section). Low (**a**,**d**) and high (**b**,**e**) LDR images and rHDR (**c**,**f**) images. Emphasis of the extended range at reduced scale is shown within the dashed box in Supplementary Fig. 27. (**g**,**h**) Actin fluorescence intensity along the profile indicated in **d** for for both LDRs, rHDR (**g**) and HDR (**h**) images. Scale bar (**a**), 100 μm. Scale bar (**d**–**f**), 20 μm.

After proof-of-principle validation, we applied our technique for two-photon high-resolution HDR imaging. We utilized a mixture of beads consisting of three different concentrations of fluorophore with fluorescence brightness spanning several orders of magnitude (‘Methods' section, Fig. 3a–j). Images (Fig. 3a–c), histograms (Fig. 3d–f, Supplementary Fig. 9) and intensity profiles (Supplementary Fig. 10) show that two-photon HDR-LSM greatly enhances the dynamic range providing information of the dim beads without losing information from the bright beads (Fig. 3g). We then compared this result to image averaging (Fig. 3h), a common approach to improve SNR. Averaging provides only a modest improvement in SNR, resulting in insufficient SNR and loss of structural information (Fig. 3i,j). HDR, however, significantly improves SNR and maintains structural information for various parameter sets (Supplementary Fig. 11). The technique was also validated on the biological samples using BS-C-1 cells stained for actin (Fig. 4a–f, ‘Methods' section). Here a large intra-scene dynamic range is present and both rHDR images (Fig. 4c,f), rHDR and HDR intensity profiles (Fig. 4g,h), reveal structural information over an extended dynamic range with enhancement near saturated pixels. To demonstrate that the information present in the rHDR images does not arise as a result of reconstruction artifacts, a comparison was performed between an HDR reconstruction obtained by acquiring images at a reduced bit depth of the PMT's dynamic range and one acquired utilizing the full dynamic range (Supplementary Fig. 12).

### Brain imaging

**Figure 5:**
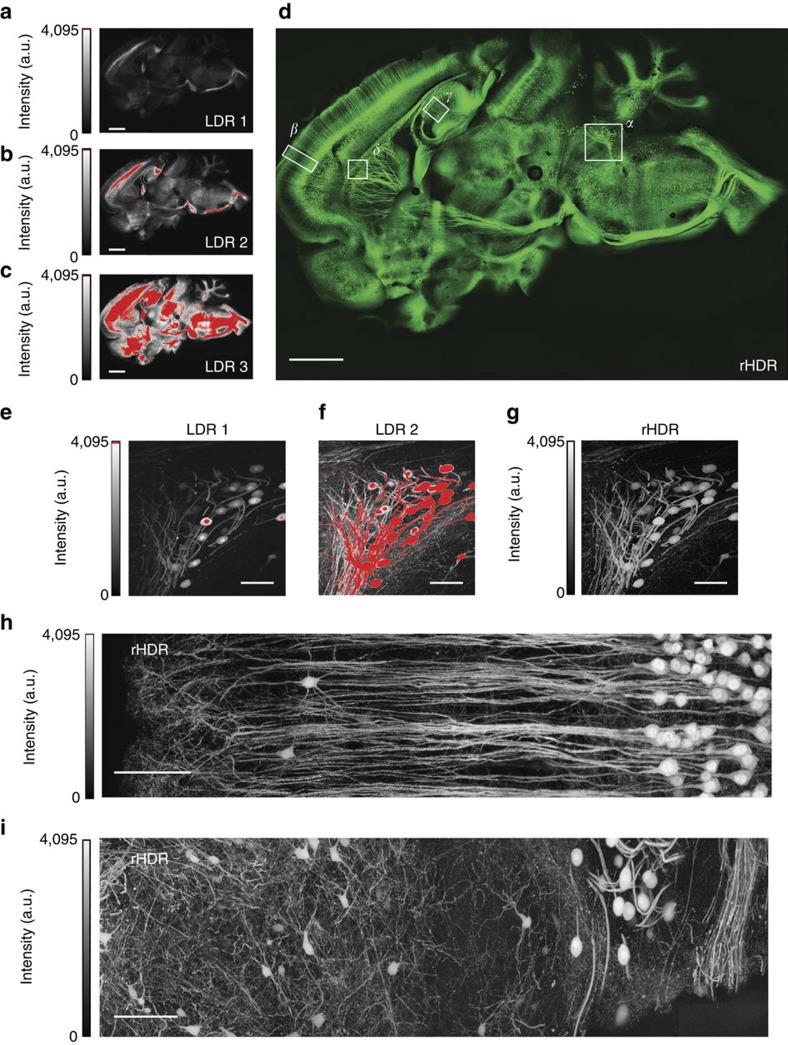
Brain HDR two-photon and confocal imaging. (**a**–**c**) Low dynamic range (LDR) images of a whole brain section with (**d**) the corresponding computed remapped HDR image (rHDR, compressed dynamic range for visualization). Red, saturation. Magnified LDRs (**e**,**f**) and rHDR (**g**) maximum intensity projection (MIP) images of the boxed region α in **d**. (**h**,**i**) Magnified rHDR MIP images of the boxed region β in **d** and the boxed region of Supplementary Fig. 15a. (**a**–**d**) Scale bar, 250 μm. (**e**–**g**) Scale bar, 100 μm. (**h**) Scale bar, 200 μm.(**i**) Scale bar, 150 μm.

**Figure 6:**
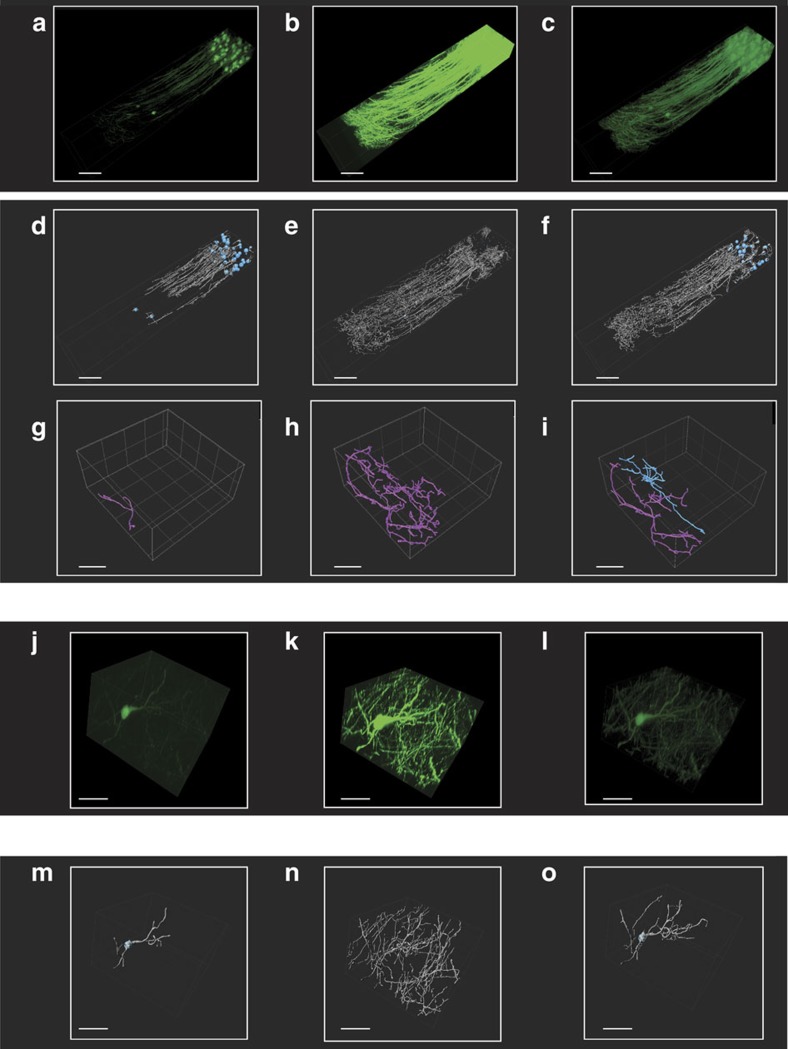
Brain HDR two-photon imaging facilitates neural segmentation. Volumetric LDRs (**a**,**b**) and rHDR (**c**) reconstructions of the boxed region β of Fig. 5d. (**d**–**f**) 3D segmentations of the different cell populations present in **a**–**c**. LDRs (**g**,**h**) and HDR (**i**) automatic 3D segmentation of two adjacent cells (blue, purple) within the boxed regions η of Supplementary Fig. 15. Volumetric LDRs (**j**,**k**) and rHDR (**l**) reconstructions of the boxed region θ of Supplementary Fig. 15. LDRs (**m**,**n**) and HDR (**o**) automatic 3D segmentation of the cell in **j**–**l**. White, dendrites and processes. Blue, cell bodies. (**a**–**f**) Scale bars, 100 μm. (**g**–**o**) Scale bars, 50 μm.

**Figure 7:**
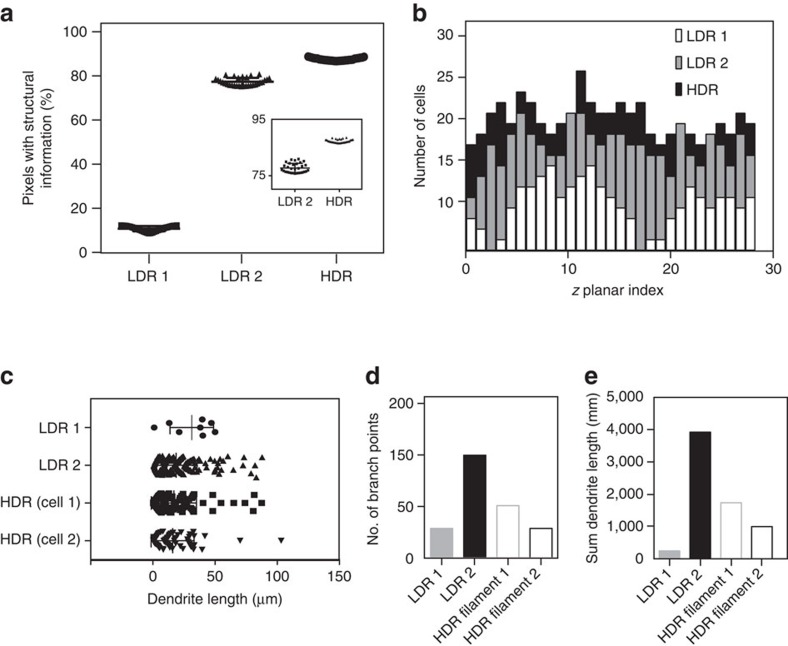
Structural information in HDR two-photon imaging brain reconstructions. (**a**) Number of pixels containing structural information in the LDRs and HDR images of the boxed regions δ and γ, respectively of Fig. 5d. LDR1, black square; LDR2, black triangle; HDR, black circle. (**b**) Number of counted cells as a function of depth within the LDRs and the respective HDR 3D volumes. Dendrite length (**c**), number of branch points (**d**) and sum of the dendrites length (**e**) calculated for the two cells of Fig. 6g–i, for both LDRs volumes, and the two HDR segmented cells.

We then utilized two-photon and confocal HDR-LSM for brain imaging (Fig. 5a–i, ‘Methods' section). Remapped HDR images (Fig. 5d,g–i, and Supplementary Figs 13–15) and three-dimensional (3D) rHDR data sets (Supplementary Fig. 16, Supplementary Movie 1, 2, 3) were used for visualization and qualitative assessments. Filament Tracer, a module of the commercial software Imaris (Bitplane, St Paul, MN, USA) developed for the detection of neurons, microtubules and filaments in 2D and 3D, was used for segmentation due to its widespread utilization in the scientific literature and the protocols availability (for example high resolution circuit mapping and phenotyping or dendritic spines segmentations)^25^,^26^. The demonstrated increased accuracy in segmentation and quantification (Supplementary Fig. 17) is attributed to the improved SNR and extended dynamic range within the composite and remapped HDR images (Fig. 6). This resulted in a lack of the common artifacts present within LDR images, including noise-induced fragmentation and saturation-induced proximity cell fusion. Moreover, our results show that HDR imaging reveals structures previously unattainable within a single acquisition (Fig. 5e–g, Supplementary Fig. 18). The composite HDR images were used for quantitative measurements of neural structures (Fig. 7a–e) as they best showcase fluorescence signal over an extended dynamic range.

### Improved image segmentation

**Figure 8:**
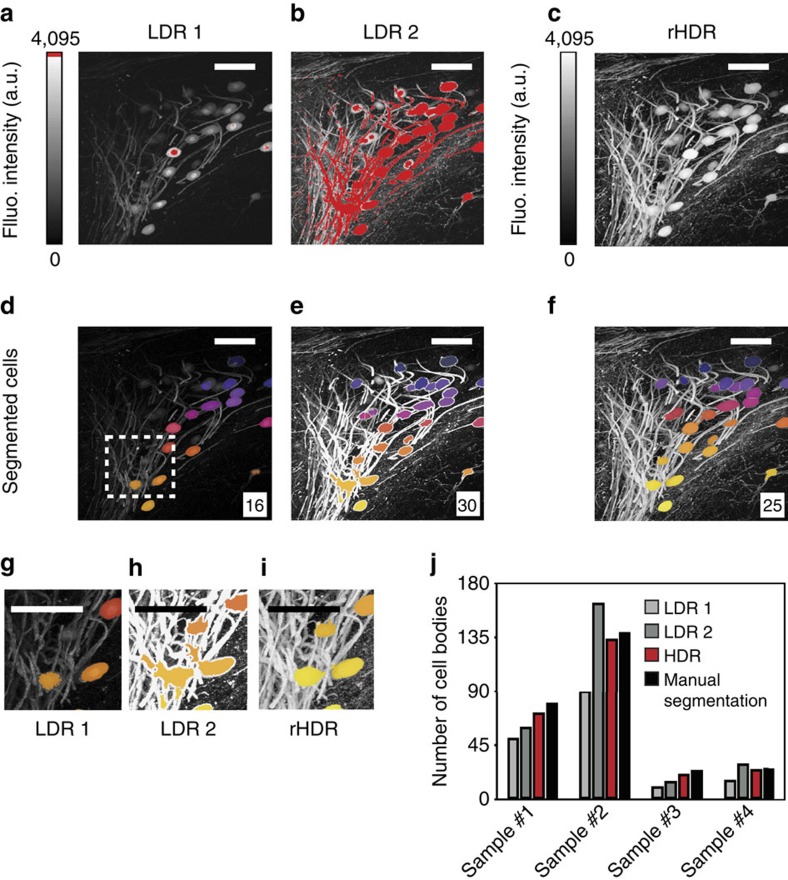
Cell body segmentation. HDR imaging allows for accurate quantification of cell bodies in sparsely populated fixed tissue specimens. LDRs (**a**,**b**) and rHDR (**c**) images of the neural cells shown in Fig. 5d (region α). (**d**–**f**) The cell bodies were segmented for each LDR and HDR image using a trainable Weka algorithm (see ‘Methods' section). In each image is indicated the number of body cells identified by the automatic segmentation algorithm. (**g**–**i**) Magnified image of the box area shown in **d**. Colours are used to help to visualize and distinguish among the different cell bodies present within the field of view. (**j**) Comparison of segmentation performance (defined as total number of cell bodies detected) between LDRs and HDR images over four different images areas. Manual segmentation and counting is used to establish the ground truth. The barplots demonstrate the improved performance of the automatic segmentation algorithm when applied to the HDR images, compared with LDR image, versus manual segmentation and counting. Image colour bar: red saturation levels. Scale bars, 100 μm. Fluo., fluorescence.

We also used a trainable Weka (Waikato Environment for Knowledge Analysis) segmentation algorithm (see ‘Methods' section), which has been demonstrated in a range of imaging pipelines for many different imaging modalities, including two-photon microscopy. The results of the segmentation approach, including segmentation of cell bodies across different regions of the brain presenting distinct degrees of cell densities, is shown in Fig. 8 and Supplementary Fig. 19. To determine the improvement in performance of the segmentation approach across the different images, a direct comparison was made between automatic and manual (here used as a reference) segmentation approaches applied to both the LDR and HDR images. Higher accuracy was achieved using the automated segmentation algorithm when applied to the HDR images rather than the LDR images (Fig. 8). Specificity, sensitivity and accuracy of cell detection were computed based on the number of false positives (that are incorrectly classified as cell bodies), false negatives (that are undetected cells) and the total number of cell bodies (Supplementary Fig. 19).

### Confocal HDR imaging for different sizes and anatomy and complexity

In addition to cellular imaging and segmentation of brain samples, we addressed the imaging and quantification of reconstruction performance of the HDR imaging platform using samples with different sizes, anatomy and complexity. First, we focused on subcellular HDR imaging of mitochondrial structures presenting a high degree of morphological complexity. Recent evidence has illustrated that mitochondria are dynamic networks, which rapidly and continuously remodel themselves^27^. Owing to their morphological complexity, attempts to study mitochondrial networks and their morphology *in vitro* have led to emerging image processing techniques to segment mitochondria labelled with fluorescent dyes or genetic reporters. Unfortunately, highly heterogeneous fluorescent expression found in many reporters affects the overall image quality. Standard LDR images (Supplementary Fig. 20) often contain cells with saturated signal or signal below or near the detection limit (that is, low SNR), making it impossible to accurately segment mitochondrial features. By combining previously validated algorithms^28^ (see ‘Methods' section), we performed segmentation on both LDR and remapped HDR images and demonstrated the ability to identify and accurately segment a larger percentage of mitochondria in rHDR images compared with LDR images (Supplementary Fig. 20).

After imaging at the subcellular level, we also tested our imaging platform and reconstruction algorithm at the macroscopic level by imaging the vasculature network in cleared organs, including the brain and the heart. The cerebral vascular structure is of fundamental importance in several brain-specific pathologies, such as glioblastoma where vessels are tortuous and disorganized and present large diameters and thicker basement membranes^29^. In the heart, the vascular network also plays a critical role in the delivery of oxygen and nutrients to the cardiomyocytes. A better understanding of the coronary network dysfunctions caused by coronary artery disease, or vascular remodelling of the endocardium following cardiac infarction is required to study disease progression. Therefore, the ability to perform high fidelity imaging and quantifications of the vascular network in these organs is in great need.

**Figure 9:**
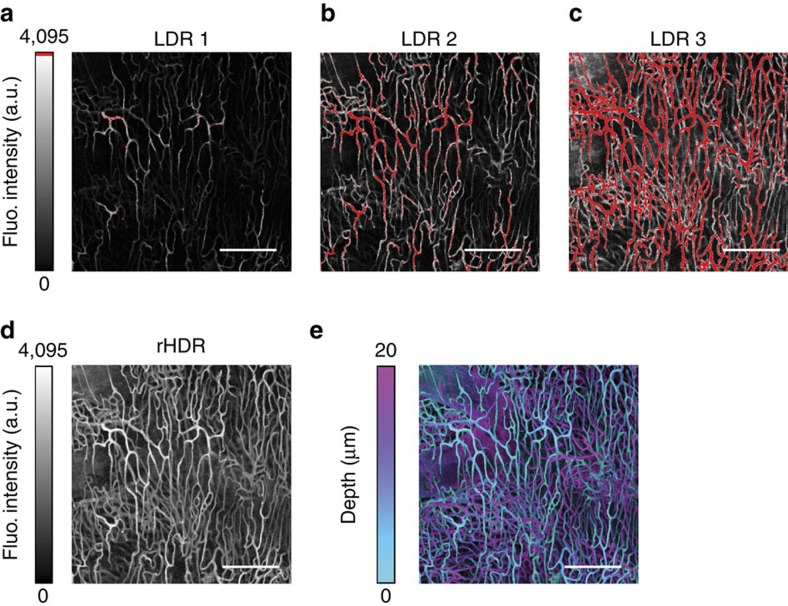
Volumetric vasculature HDR confocal imaging. LDRs (**a**–**c**) Images of a cleared Dil stained heart, and (**d**) corresponding rHDR image reconstruction. (**e**) Projection of the three-dimensional rHDR acquisition of the vasculature where colours represent different imaging depths and brightness is related to the fluorescence (Fluo.) signal amplitude. Scale bar, 150 μm.

Following Dil staining (see ‘Methods' section), we imaged the cleared brain (Supplementary Fig. 21) and heart (Fig. 9) using both LDR and HDR imaging. We then quantified features of the vasculature network, including the number of vascular branches in the heart (Fig. 9, Supplementary Fig. 22). Automated segmentation of rHDR images allowed for the identification of vascular features that agreed with values obtained using ground truth manual segmentation. Conversely, LDR image segmentation resulted in a high degree of vasculature fragmentation (low branch length) due to the low SNR present within the image.

### *In vivo* real-time two-photon HDR imaging

To highlight real-time acquisition capabilities of our HDR imaging platform, we then performed real-time two-photon HDR microscopy for longitudinal imaging in the presence of physiological motion and for quantification of *in vivo* fast tracer kinetics during functional imaging. Imaging was performed in the subcutaneous tissue of mice implanted with a dorsal window chamber (see ‘Methods' section). During intravital microscopy imaging, both cardiac and respiratory cycles compromise the ultimate spatial and temporal imaging resolution. If the images are acquired sequentially, physiologically induced motion-artifacts degrade the quality of the HDR reconstructed images. Different areas of sequentially captured images may be misaligned in consecutive frames, giving rise to severe ghosting artifacts in the final reconstructions. In the real-time acquisition modality these artifacts do not occur as the pixels used in the HDR reconstruction are acquired simultaneously via multiple PMTs (Supplementary Fig. 23).

Another important application of real-time HDR microscopy is the possibility to obtain *in vivo* accurate quantitative assessments of the time intensity variations that represent the kinetics of a probe across multiple tissue compartments. This is particularly relevant for studying the intravascular extravasation and extravascular pharmacokinetics of fluorescently labelled drugs. Single-cell analysis of drug pharmacokinetics requires the ability to quantify drug concentration kinetics in the vascular, interstitial and cellular compartments^30^. However, conventional LDR microscopy imaging does not have sufficient dynamic range to handle the substantial spatio-temporal variations in drug signal intensity, making it challenging to quantify drug pharmacokinetics at the single-cell level^30^. As a proof of concept, we characterized the vascular kinetics following tail vein injection of a bolus of different molecular weight FITC-Dextrans, in a dorsal window chamber mouse model.

**Figure 10:**
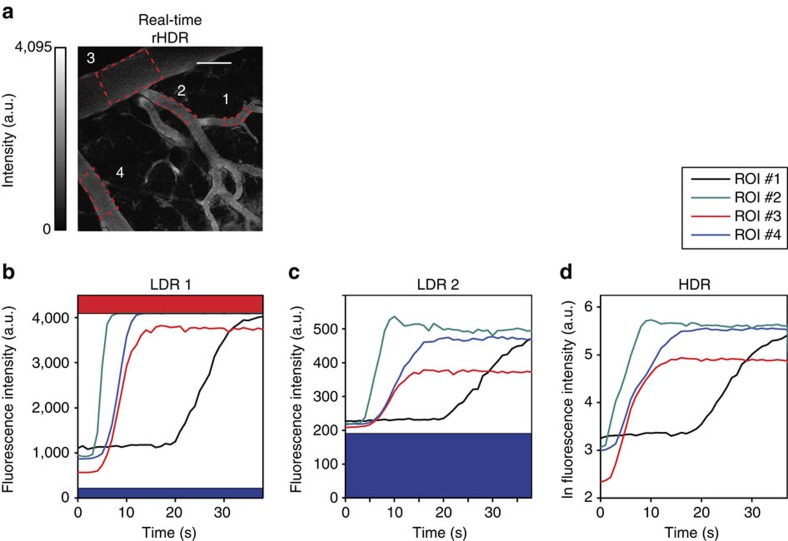
*In vivo* intravascular dye kinetics. *In vivo* intravascular real-time quantification of the time–intensity variations demonstrating the vascular pharmacokinetics of a fluorescent probe across multiple regions of interests (ROIs). A bolus of 2 MDa FITC-Dextran was injected intravenously through the lateral tail vein (see ‘Methods' section) and vascular kinetics were captured by collecting a time sequence of real-time HDR images. (**a**) ROIs were selected in several blood vessels within a dorsal window chamber and time–intensity curves calculated as the mean of the signal within the ROI as a function of time. (**b**–**d**) Time–intensity curves are plotted for both (**b**,**c**) LDR and (**d**) HDR time sequences. LDR sequences have a limited ability to capture the full dynamic range of intensities, while HDR sequences present a high SNR and extended dynamic range demonstrating the ability to maintain imaging fidelity for kinetic quantification. Scale bar, 125 μm.

A bolus of 2 MDa FITC-Dextran was injected intravenously followed by a bolus of a 4 kDa FITC-dextran (see ‘Methods' section). The temporal resolution of the two-photon real-time acquisition was sufficient to capture the vascular kinetics of the 2 MDa probe (Fig. 10) and the extravasation of the 4 kDa probe into the interstitial tissue (Supplementary Fig. 24). Regions of interest were selected in the vascular (Fig. 10) and extravascular (Supplementary Fig. 24) compartments and time–intensity curves were calculated as the mean of the signal within the region of interest as a function of time.

Signal degradation due to photobleaching during HDR imaging, under typical acquisition conditions, was not observed for all the probes used in this study (Supplementary Fig. 25). This was also valid for the cells expressing a green fluorescent protein (GFP) genetic reporter of mitochondria, cells stained with AlexaFluor-488 Phalloidin, Dil stained vasculature in both fixed and cleared tissue, and brain tissue sections stained with AlexaFluor-488 conjugated secondary antibodies. AlexaFluor dyes are frequently used in cleared samples for whole-organ imaging, due to their low photobleaching and for their stability in clearing solution over periods of several months and over multiple imaging sessions^31^. Lipophilic tracers such as Dil also exhibit low photobleaching and high fluorescence intensity making them suitable for laser scanning microscopy in general as well as for imaging in cleared organs^32^.

## Discussion

The recent introduction of innovative high-throughput and high-resolution imaging modalities along with the concurrent development of novel clearing techniques^33^,^34^,^35^,^36^,^37^ enables sectioning-free imaging of intact brain tissue^38^ facilitating mapping of neural connectivity (connectome) of the whole brain at the microscopic level^33^. However, data analysis constitutes the major bottleneck of the analysis pipeline and requires the use of sophisticated unsupervised image-processing techniques for automatic 3D digital reconstruction and tracing of the individual neuron processes. Unfortunately, the presence of a wide range of signal intensities is a common challenge in neuronal imaging, particularly for large specimens such as the entire brain. Some neural processes are extremely fine and difficult to visualize with fluorescent proteins, requiring scans at high laser power or high gain to resolve their structure. In contrast, larger dendritic varicosities (sites of synaptic contact) often are robustly labelled with fluorescent proteins, requiring lower laser power or gain to preserve structural details (Supplementary Fig. 26). At the microscope level, we are therefore typically forced to strike a balance in the scanning parameters, and it is common to observe loss of data within and among cells (Supplementary Fig. 27), resulting in reduced segmentation accuracy.

Here we have shown that two-photon and confocal HDR-LSM enable visualization and quantification of dim and bright structures within the same field of view, via significantly improved SNR and extended dynamic range. In addition to providing more aesthetically pleasing images through HDR remapping, our HDR fusion algorithm provides more accurate measurements of fluorophore concentration. Moreover, the simultaneous acquisition of multiple LDR images enables real-time HDR-LSM, where non-stationary objects could be imaged in real-time making the technique also suitable for *in vivo* imaging. Specifically, we have illustrated that real-time two-photon HDR imaging provides the ability to remove artifacts caused by physiological motion, to capture data with sufficient temporal resolution to image the tracer kinetics in real-time, and has sufficient dynamic range to capture the substantial signal variations observed between the vascular and extravascular compartments. This demonstrates that real-time two-photon HDR-LSM is particularly useful for *in vivo* systematic analysis of fluorescent drug pharmacokinetics across tissue compartments, and between heterogeneous cell populations in real time^30^,^39^,^40^.

Moreover, the real-time HDR acquisition allows for accelerated acquisition of large data sets with large dynamic ranges (Supplementary Fig. 8) preventing lengthy imaging sessions. This is particularly relevant for cleared tissue where hours or days are typically spent for whole-organ imaging with thousands of optical sections collected per single position, in composite stitched images that can cover areas across 1–2 cm in area (approximately one million images per sample).

Compared with other HDR approaches, our technique is simple to implement in any commercially available two-photon imaging system and/or confocal microscope (Supplementary Figs 2–4), at virtually zero cost, and thus can be widely adopted. In addition, our HDR imaging approach may be easily extended to other microscope configurations, including light-sheet, wide-field and spinning-disk microscopy.

We envision a number of other applications where both real-time and sequential two-photon and confocal HDR-LSM would be beneficial such as cell-to-cell communication, detection of fine processes such as filopodia or tunnelling nanotubes^41^, imaging of intracellular organelles, network analysis and branching of dendritic and glial cells among others.

## Methods

### Cell culture and staining

BS-C-1 cells were obtained from ATCC and cultured in Eagles Minimum Essential Media supplemented with 10% FBS and 1% penicillin/streptomycin in a tissue culture incubator. For imaging, cells were seeded on Poly-L-Lysine (Sigma-Aldrich) coated 12-well slides (Ibidi) and cultured overnight.

The cells were fixed in 4% paraformaldehyde (Electron Microscopy Sciences) for 15 min and washed for 3 × 5 min in TBS. Following fixation, the cells were permeabilized using a solution of 0.1% Triton-X in TBS and blocked for 30 min in Odyssey blocking buffer (LI-COR Biosciences). The cells were then incubated with AlexaFluor-488 Phalloidin (Life Sciences), diluted 1:20 in phosphate-buffered saline (PBS) for 15 min and washed for 3 × 5 min in TBS. Finally, the slides were affixed with coverslips before imaging.

### Mitochondria expressing cells

The OVCA-429 cells were transduced with a GFP genetic reporter of mitochondria using a CellLight Fluorescent Protein Labeling kit (ThermoFisher). The cells were transduced 2 days before imaging according to the manufacturer's instructions. On the day of imaging, the cells were fixed in a 4% solution of paraformaldehyde in PBS for 10 min, and sealed with a coverslip.

### Tissue section preparation

Adult Thy1-YFP-H (YFPH)1 mice were anaesthetized with pentobarbital and transcardially perfused with PBS followed by 4% paraformaldehyde (PFA) in PBS. Whole brains were dissected and post-fixed overnight in 4% PFA at 4 °C, washed in PBS, then immersed in 30% sucrose in PBS at 4 °C overnight for sectioning using a cryostat (100–500 μm). Sections were incubated with chicken anti-GFP primary antibodies (Abcam) in blocking buffer (1% horse serum, 0.1% Triton X-100, 0.05% azide in PBS) for 4 days, followed by a wash with PBS and incubation with donkey anti-chicken Alexafluor-488 conjugated secondary antibodies (Jackson Immunoresearch) in blocking buffer. Antibody incubation periods were performed at room temperature^42^.

### Vessel staining

The brain and heart vasculature were stained using a fluorescent lipophilic dye, DiIC18(3), which accumulates at high concentration in endothelial cell membrane. The staining procedure is very rapid and efficient and the resulting bright fluorescence signal is characterized by very low photobleaching^43^,^44^ making it particularly suitable for laser scanning microscopy. A protocol similar to the one indicated in ref. 32 has been followed. Briefly, after being euthanized the mouse heart was made accessible through thoracotomy and the mouse was perfused by inserting a needle into the left ventricle and with the right atrium cut open. The heart was injected with a solution consisting of 2 ml of PBS, followed by 5 ml of DiI solution and 5 ml of 4% PFA at a rate of 1 ml min^−1^. The samples were then excised, cut and imaged. To reach higher penetration imaging depth, some specimens were also fixed in 4% PFA overnight and then treated with a clearing solution allowing for whole-organ imaging.

### Optical clearing

Tissue clearing and imaging were performed using a slightly modified version of the CUBIC (clear, unobstructed brain imaging cocktails and computational analysis) method^33^, which is based on the immersion of fixed tissue in a chemical mixture containing aminoalcohols. CUBIC has been proven to enable rapid whole-brain multicolour imaging of fluorescent proteins or immunostained samples.

One-millimeter fixed brain sections were immersed in a solution obtained by mixing 25 wt% urea (Fisher Scientific, U16–3), 25 wt% *N*,*N*,*N*′,*N*′-tetrakis(2-hydroxypropyl) ethylenediamine (Fisher Scientific 50-014-48142), and 15 wt% Triton X-100 (Life Technologies, 85111). Sectioned slices remained immersed for 2 days at 37 °C, while gently shaken. The cleared slices were then mounted on a custom-made sample holder for microscopy imaging. Alternatively, tissue stained with DiI were cleared using Rapiclear 1.49 (ref. 45), a clearing agent compatible with various endogenous fluorescence proteins and lipophilic tracers such as DiI, following overnight immersion in solution.

### Two-photon microscope configuration

The two-photon microscopy setup, illustrated in Fig. 1b and in Supplementary Figs 2 and 4 is based on a custom modified Olympus FV1000-MPE (Olympus, USA) laser scanning microscopy system equipped with an upright BX61-WI microscope (Olympus, USA). Excitation light (red beam) from a Ti:sapphire laser is focused onto the imaged sample with a × 25 1.05 NA water immersion objective (XL Plan N, 2 mm working distance) or a × 25 1.00 NA ScaleView immersion objective (XL Plan N, 4 mm working distance). The emitted fluorescent light (green beam) is epi-collected through the same focusing objective and reflected by a dichroic filter, DC, (690 nm) toward a non-descanned detection path. After passing first through a lowpass filter, LP, (685 nm) and a bandpass filter, BP, (490–540 nm), the fluorescent light is split into two beams, IF1, of equal intensity by a 50/50 beam-splitter, BS1. The first component of the beam is directly detected by the first photomultiplier tube, PMT1, with no neutral density filter attenuation (IF1). Meanwhile, the second component of the beam (IF1) is split again by a second 50/50 beam-splitter, BS2, into two new components, IF2 and IF3. Beams IF2 and IF3 are detected by the photomultiplier tubes PMT2 and PMT3, respectively, after passing through two separate neutral density filters ODF1 and ODF2 presenting different optical densities. Three fluorescence signals are then acquired simultaneously and in real time with varying attenuations as determined by the three different optical density filters (typically 0, 0.9 and 1.8 dB). Depending on the range of fluorescence signal present within the samples, two channels can also be acquired instead of three. To avoid bleaching during acquisition, the laser power was always kept well below 10 mW, at typical PMT voltages of 410–650 V. Asymmetric beamsplitters can be used to maximize the number of photons collected at the detectors. A sequential imaging approach can also be implemented for two-photon microscopy, when relying on one PMT only or if accurate dynamic range tuning is desired. Also, two PMTs can be sufficient instead of three, depending on the intra-scene dynamic range, reducing the total number of acquired images.

### Confocal microscope configuration

The imaging system used for this work allows for dual confocal and multiphoton microscopy, and can be easily extended for sequential confocal laser scanning microscopy HDR as illustrated in detail in Supplementary Figs 2–4 and 28. Excitation light (blue beam) from a 473-nm diode laser is focused onto the sample using the same lens objectives utilized for two-photon imaging. The emitted fluorescent light (green beam) is epi-collected through the focusing objective and reflected by a dichroic beam splitter DC (SDM560). Light is then bandpass filtered (BA490–540) and directed toward a detection path after being reflected by the galvanometer scanner. Image acquisition at different intensity peak values, necessary for HDR processing, can be obtained by appropriately selecting the excitation laser power or by changing the PMT voltage values, which determine the level of signal amplification. To provide high-quality images, the laser power and PMT voltage should be chosen such that the sample is not bleached and high noise levels are not introduced, which would restrict the dynamic range of the acquired image.

### Image acquisition

3D data sets were all collected in optically cleared tissue sections. Z stacks were collected for both confocal and two-photon microscopy using two motorized stages controlling both planar and axial translations. Typical data set acquisition consisted of two to four z stacks with approximately 100 optical sections (1 μm per section). First, whole-brain HDR images were obtained in confocal mode using a × 10 (UMPLFL, 0.3 NA, 10 mm WD) or alternatively a × 2 air objective (XLFluor, 0.14 NA, 21 mm WD). These preliminary measurements allow for improved identification of interesting structures. For confocal microscopy, the images are collected sequentially by varying the intensity of the excitation light. For real-time two-photon microscopy, optical density filters, or asymmetric beam splitter(s) are added before the detectors (Fig. 1b). The excitation power and voltage were chosen to minimize noise and photobleaching.

### Validation phantom experiment

For proof of principle and HDR validation (Fig. 2, Supplementary Fig. 6), fluorescein was prepared with 1:1, 1:10 and 1:100 dilutions in DI water.

### Beads sample experiment

For structural validation (Fig. 3), 2.5 μm green-fluorescent microspheres (LinearFlow Flow Cytometry Intensity Calibration Kit, 488 nm excitation/515 nm emission, Life Technologies). The kit's microspheres, originally intended for flow-cytometry calibration, have varying discrete values of fluorescence intensity. Microspheres with 0.3, 3 and 100% relative intensity (bead populations 1, 2 and 3 respectively) were selected and mixed together in equal parts. The beads were then deposited on a glass slide with a cover slip and sealed before imaging. Within each population, the intensity distribution is highly homogenous.

### Neural segmentation

High-content analysis in neuroscience makes use of several image-processing tools and resources for digital tracing^46^ and all existing software requires a certain amount of user intervention^47^. Within the neuroscience community, Neurolucida, Amira, Eutectic NTS, NeuronJ and Filament Tracer are the most popular software packages and provide similar functionality.

Neural segmentations shown in Fig. 6d–h and Fig. 7c–e were performed with IMARIS filament tracker for automatic detection. Automatic segmentation was chosen over manual segmentation to prevent bias. The parameters were constant when comparing raw LDR images to high dynamic range images. User input parameters were: local contrast threshold=5; signal threshold=mean noise±3σ; starting point diameter=20 μm, ending point diameter=2 μm. Disconnected segments were removed. Segmentation for HDR images was done on 32-bit data.

Cellsegm^48^ was used for high-throughput 3D cell segmentation of the data presented in Fig. 7b. The Weka automated segmentation algorithm was used to identify regions with cell bodies^49^. The classifier was trained using an equal number of cell bodies from both LDR images. After locating the cell body, the mask was eroded, and a watershed filter was used to separate the neighbouring cell bodies. The cell bodies were counted using a 3D particle counting method previously described by Bolte and Corelieres^50^. The algorithm was used to segment data presented in Fig. 8, and Supplementary Fig. 19.

### Mitochondrial segmentation

Mitochondria were segmented by combining previously used algorithms^28^. Briefly, a ‘rolling ball' background subtraction was first conducted on the images. To prepare the images for segmentation, a modified version of the previously published algorithm by Giedt *et al*.^28^ was then applied. This consisted of applying a convolution filter with [5 × 5] matrix with a centre element of 24 and the remaining ones equal to −1, followed by Fast Fourier Transform-based bandpass filtering. A maximum entropy-based thresholding algorithm was applied and size-based filtering was used to eliminate small objects. All analyses were conducted using Matlab. The algorithm was used to segment data presented in Supplementary Fig. 20.

### Vasculature segmentation

Vasculature segmentation was performed using a machine learning method based on a trainable Weka segmentation algorithm that has been demonstrated on a wide range of imaging applications and for many different imaging modalities^49^, including two-photon microscopy. The algorithm was used to segment data presented in Supplementary Fig. 22.

### Animal model

The animal experiments were performed in accordance with the Institutional Animal Care and Use Committee at Massachusetts General Hospital. The surgical procedures were conducted under sterile conditions and facilitated with the use of a zoom stereomicroscope. The mice were anaesthetized by isoflurane vaporization (Harvard Apparatus) at a flow rate of 2 l min^−1^ isoflurane: 2 l min^−1^ oxygen while their body temperature was kept constant at 37 °C during both surgical procedures and imaging experiments. The dorsal skinfold window chambers were implanted 2 days before imaging following a well-established protocol. Because the experiments involved studies of vascular perfusion, it is crucial to insert a metal spacer within the dorsal window chamber to prevent excessive compression of both tissue and vessels.

### *In vivo* imaging of vascular perfusion and extravasation

The mice were anaesthetized as described above with an isoflurane rate of approximately 1 l min^−1^. A custom stabilizer plate was used to secure the dorsal skinfold window chamber and reducing motion artifacts and axial drifts during imaging. The animals were kept warm with a heating plate to keep their temperature constant at 37 °C. A catheter was inserted into the tail vein and was used for bolus administration of fluorescent dextrans. Two fluorescent probes (fluorescein isothiocyanate-dextran, FITC-Dextran) with different molecular weights (2 MDa and 4 kDa) were used as a fluorescent probe to study vascular perfusion and extravasation. A bolus of 2 MDa FITC-Dextran was injected intravenously (2.5 μM) through the lateral tail vein, followed by a bolus of a 4 kDa FITC-dextran (1.2 mM). Because the perfusion kinetic is very fast, it is important to capture the fluorescence signal at the moment of injection leaving no time to adjust the focal plane. Therefore, a third dye (TRITC-Dextran 4 kDa) at a different emission channel was first used to find a representative imaging area and the correct imaging plane. Two-photon imaging of FITC (excitation 790 nm, emission 490–540 nm) was performed using a × 25 water immersion objective. TRITC was imaged using the HDR confocal microscopy method previously described. A × 2 objective was used to initially identify the best imaging area within the window chamber.

HDR acquisition with real-time reconstructions was performed in free running mode at a 2 Hz frame rate to capture perfusion and extravasation dynamics of the FITC-Dextran. To maximize fluorescence collection efficiency, 90/10 beam splitters were used instead of attenuating filters (Supplementary Fig. 4).

## Additional information

**How to cite this article:** Vinegoni, C. *et al*. Real-time high dynamic range laser scanning microscopy. *Nat. Commun.* 7:11077 doi: 10.1038/ncomms11077 (2016).

## Supplementary Material

Supplementary Information Supplementary Figures 1-28, Supplementary Notes 1-3 and Supplementary References

Supplementary Movie 1 Low intensity 3D rendering of pyramidal neurons

Supplementary Movie 2 High intensity 3D dataset of pyramidal neurons.

Supplementary Movie 3 rHDR 3D dataset of pyramidal neurons.
