# Supplementary material for: Real-time high dynamic range laser scanning microscopy
Source: Nat Commun. 2016 Apr 1;7:11077. doi: 10.1038/ncomms11077 (PMC4821995; doi:10.1038/ncomms11077)
Supplement: Supplementary Information — Supplementary Figures 1-28, Supplementary Notes 1-3 and Supplementary References [file ncomms11077-s1.pdf]

Supplementary Figure 1

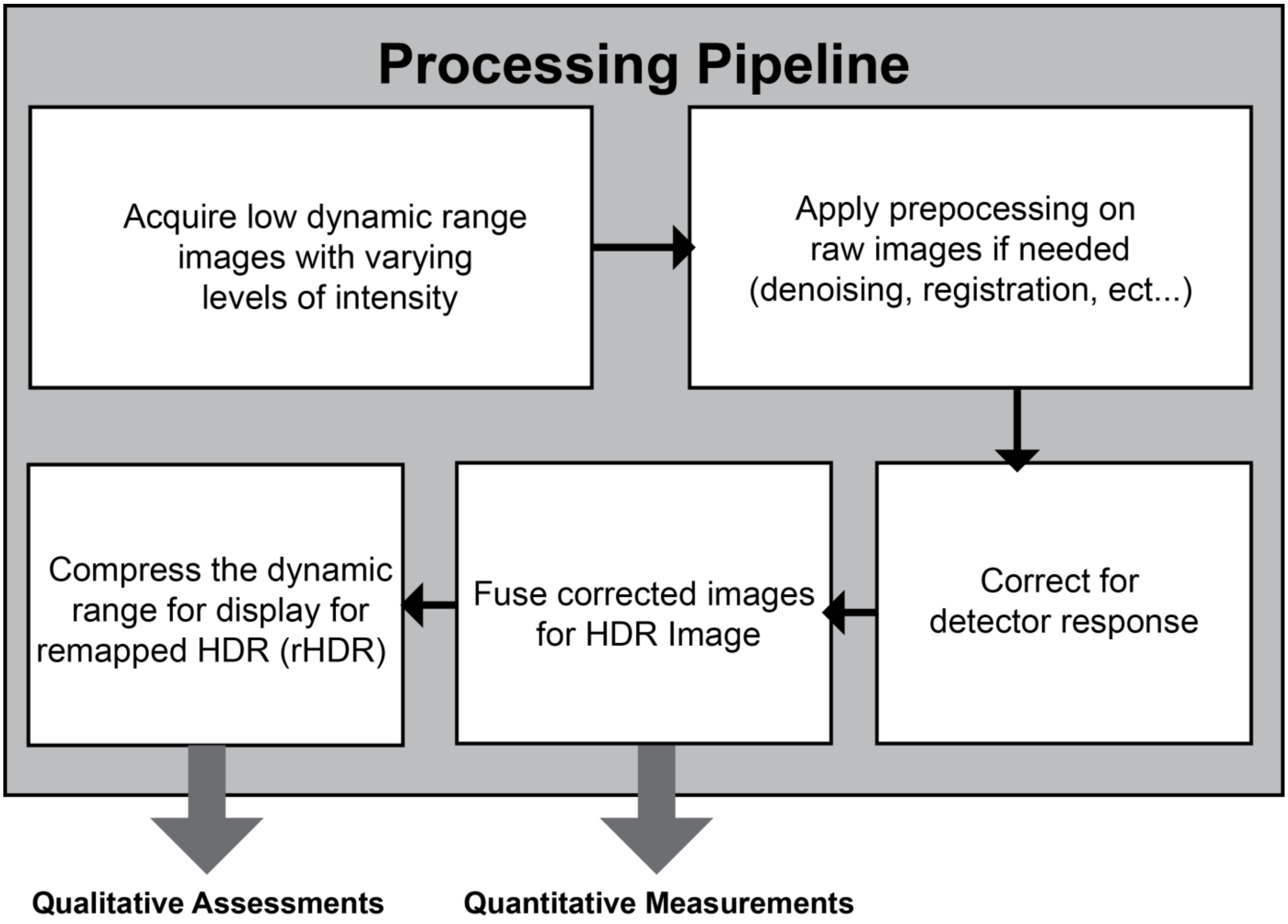

Processing pipeline for laser scanning microscopy HDR imaging.
The processing pipeline consists of five distinct phases. Images are first acquired after determining the appropriate filter densities or excitation power. For real-time 2-photon microscopy, images are collected simultaneously and processing occurs in real-time. For confocal microscopy, data are collected sequentially and processing occurs at the end of the acquisition. Data can then be preprocessed for denoising or registration if needed. Denoising was not used in this work. Images are then corrected for the detector response (Supplementary Fig. 5, Supplementary Note 3) and a final HDR image is reconstructed. At this point the HDR image (64-bit or 32-bit format) is ready to be processed for segmentation or cell counting. Finally, the HDR images are remapped to a lower dynamic range (16-bit, 12-bit, or 8-bit) to generate rHDR images for display purposes.

Supplementary Figure 2

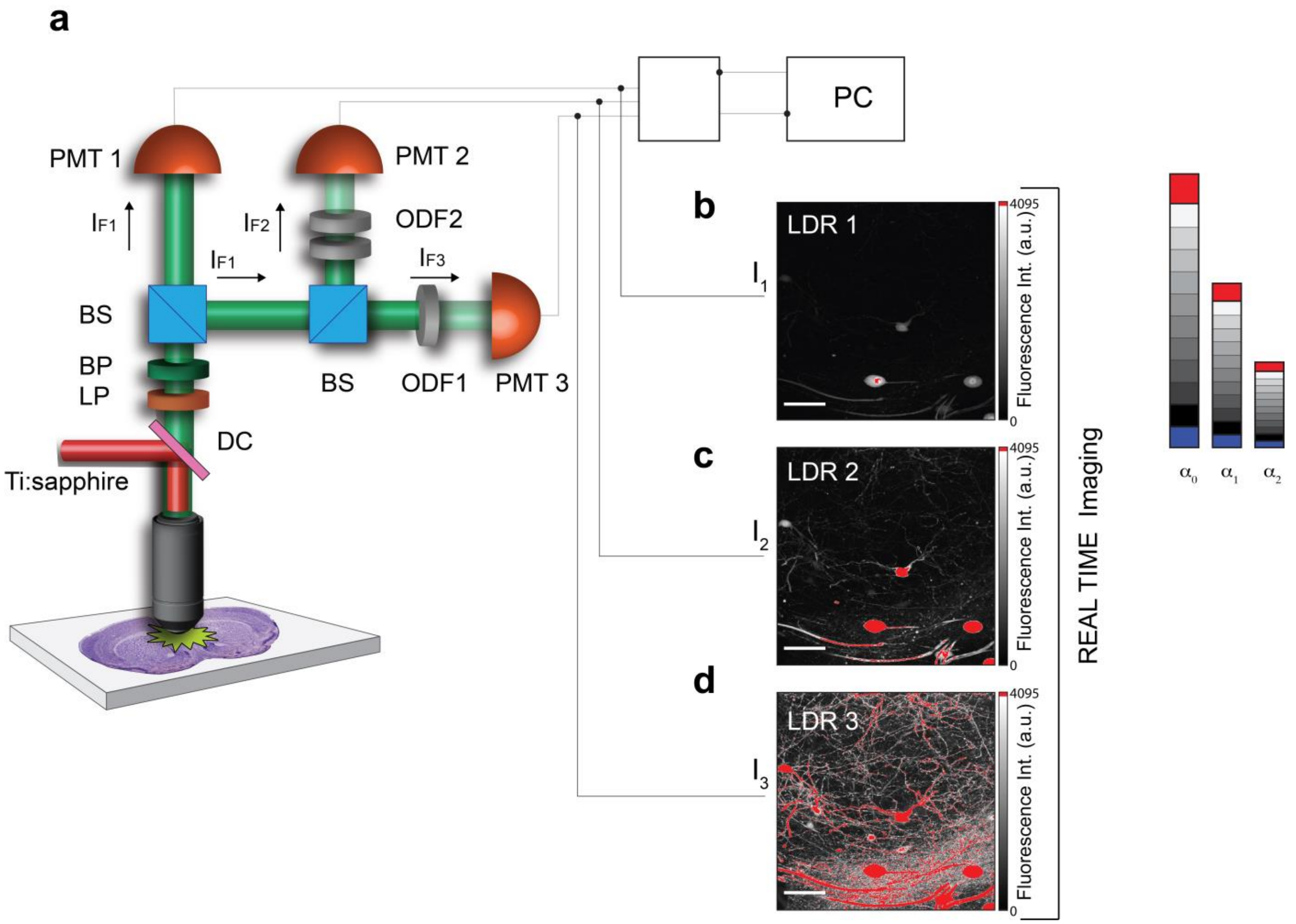

Schematic representation of the two-photon imaging setup for real-time HDR imaging. (**a**) BS beam splitter, BP bandpass filter, LP longpass filter, DC dichroic mirror, ODF neutral optical density filter, PMT photomultiplier tubes. (**b-d**) Three low dynamic range images (LDR1, LDR2, LDR3) are simultaneously acquired by the three PMTs. The different absorption values of the neutral optical density filters (ODF_i) present in front of the detectors attenuates the light by a factor α_i (Fig. 1a) and sets, for each image, the mid level of the dynamic range where each PMT is centered. Because the acquisition is performed with three distinct PMTs, the LDR images are acquired simultaneously. Depending on the range of fluorescence signal present within the samples, two channels can be also acquired instead of three. In this case the fluorescence is distributed by only one beam splitter in equal ratios (50%, 50%) between the two PMTs, and subsequently attenuated with different ratios using ODFs. Image color bar: blu, dark noise; red, saturation levels. Scale bar, 100 μm.

Supplementary Figure 3

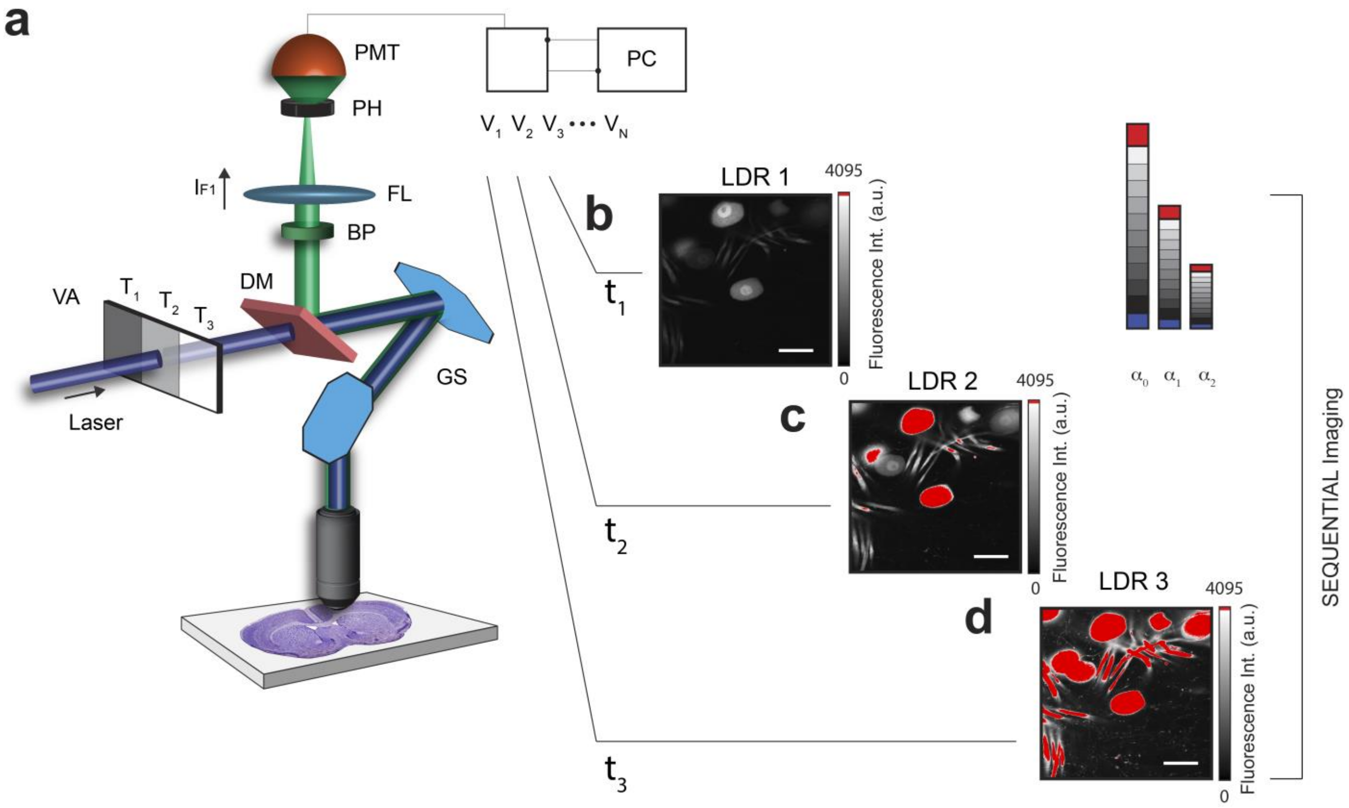

Schematic representation of the confocal imaging setup for sequential HDR imaging. (**a**) VA, variable optical density filter with transmittivity T_1,T_2, … T_n. DM dichroic mirror, GS galvanometer based scanning mirror system, BP bandpass filter, FL focusing lens, PH pinhole, and PMT photomultiplier tube. All images (**b-d**) are collected with a single detector. Acquisition occurs sequentially at three different times (t_1, t_2, t_3). Different PMT voltage settings V_1, V_2, … V_n, can be set in order to change the sensitivity of the photodetector such that the dynamic range of each single measurement is centered at a different signal level. Alternatively, the laser intensity can be changed by controlling a tunable transmissivity filter. For both situations different limitations are present. When changing the laser power, bleaching can occur creating artifacts in the reconstructed images. When changing the voltage settings of the PMT, noise background and signal noise are present in different values for each acquired image. Particular care needs to be taken to choose the appropriate imaging parameters. Image color bar: blue, dark noise; red, saturation levels. Scale bar, 40 μm.

Supplementary Figure 4

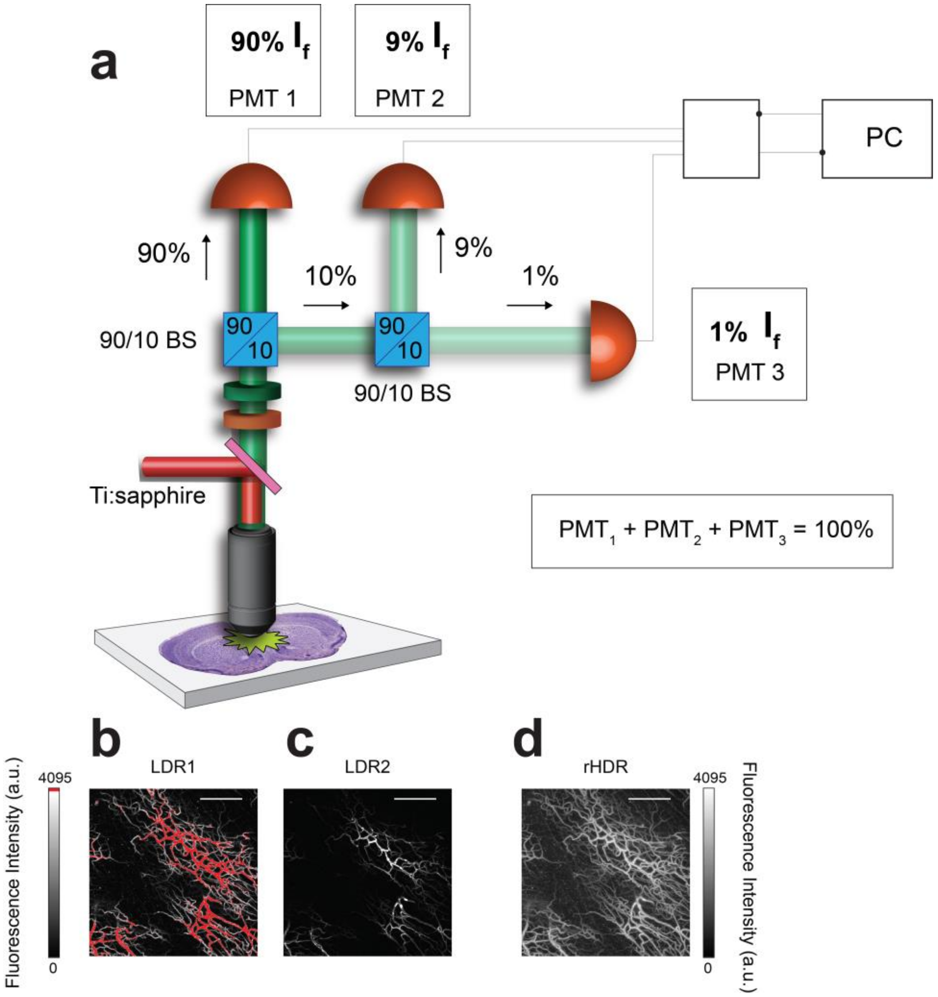

HDR two-photon imaging setup configuration using 90/10 beamsplitters.
(**a**) Schematic representation of the two-photon imaging setup for real-time HDR imaging in the configuration without optical density filters. Fluorescence signals of different intensities are captured by the PMTs using 90/10 non-polarizing beam splitters (90/10 BS) with a 90% and 10% splitting ratios in transmission and reflectivity, respectively. In this configuration no photon is lost during HDR acquisition. In fact, the whole signal contributes to the HDR image reconstruction being the fluorescence distributed by the beam splitters in different ratios (90%, 9%, and 1%) to the three PMTs. Depending on the range of fluorescence signal present within the samples, it is possible to acquire two channels instead of three. In this case the fluorescence is distributed by only one beam splitter in different ratios (90%, 10%) to the two PMTs. (**b-c**) Low dynamic range images centered at different points of the PMT's dynamic range are simultaneously acquired by the PMTs. Acquisitions are performed in real-time and rHDR images **(d)** are reconstructed. Here the vasculature of a DiI stained heart is shown. Image color bar: red, saturation levels. Scale bar, 125 μm.

Supplementary Figure 5

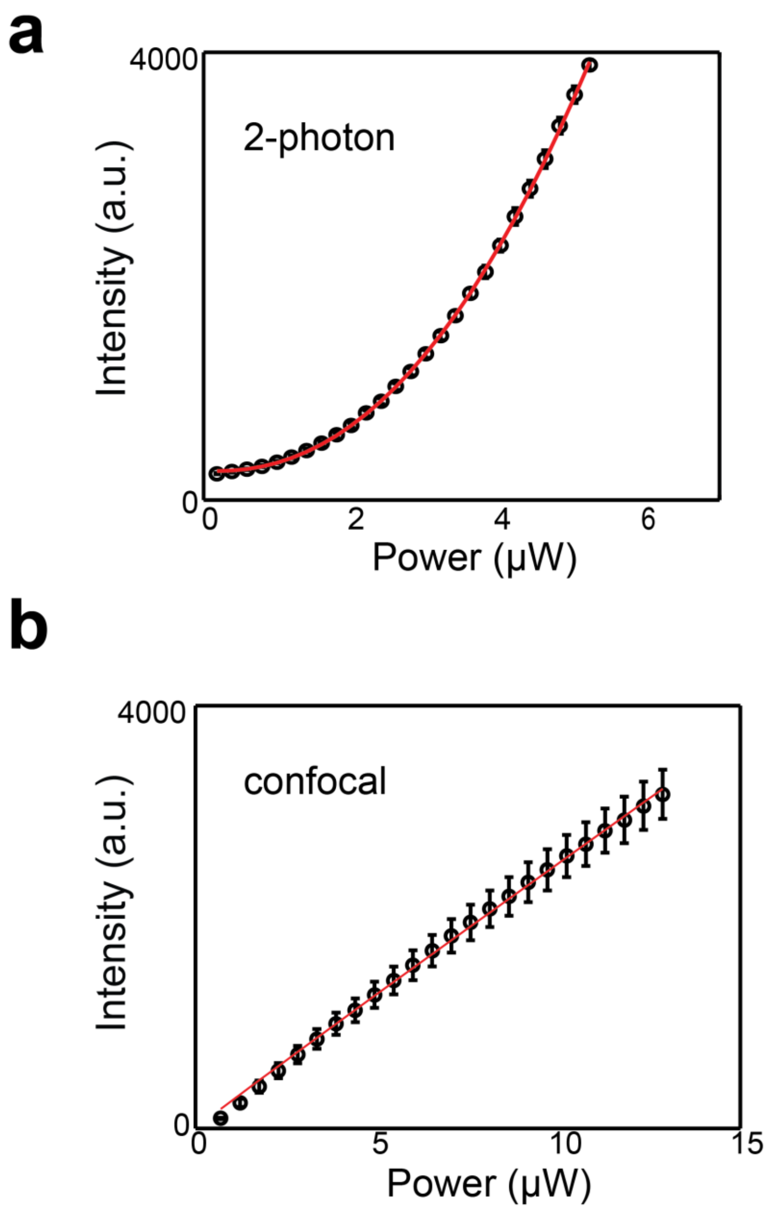

Calibration curves
Calibration curves obtained for the detectors that are used to correct the (**a**) two-photon and (**b**) confocal HDR images, after acquisition (Supplementary Note 3). The calibration curves are determined once for each PMT and voltage settings. Error bars correspond to the standard deviation of the mean, calculated on all pixels within a central region of interest of each acquired image at different powers.

Supplementary Figure 6

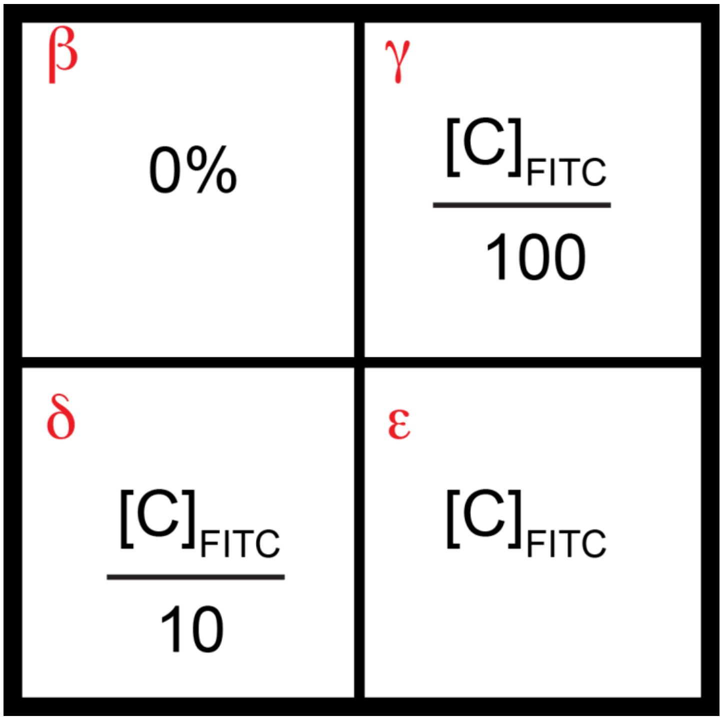

Layout of the fluorescent phantom for proof-of-principle and SNR validation.
The imaging phantom shown in Fig. 2 is composed of four distinct non-adjacent areas ($\beta,\gamma,\delta,\varepsilon$) with varying fluorophore concentrations (Online Methods) in DI water with 1:1 1:10 and 1:100 dilutions as well as background. The phantom provides a simple imaging object presenting a large dynamic range, for proof-of-principle validation and exposition.

Supplementary Figure 7

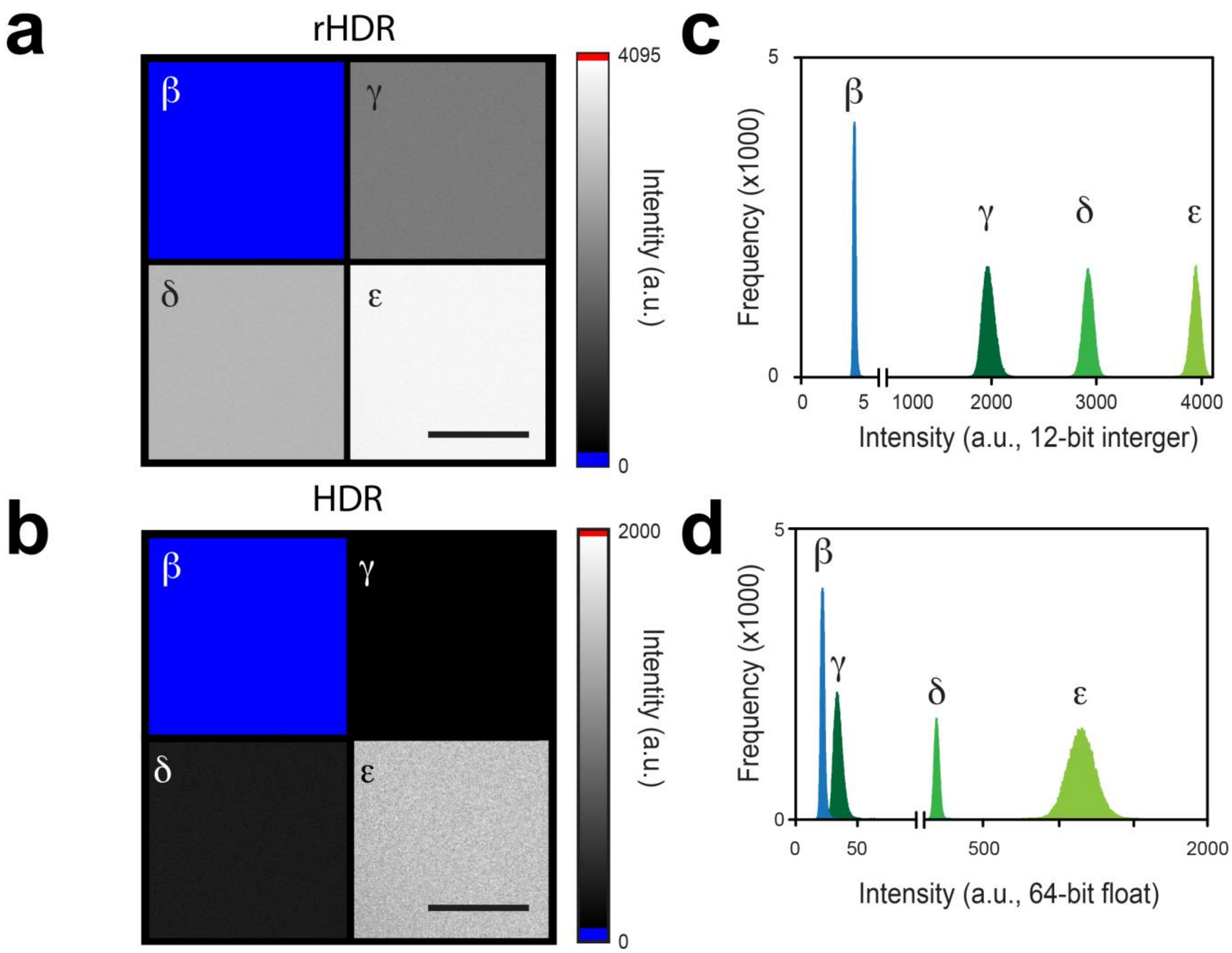

HDR versus remapped HDR images.
Comparison between the (**a**) rHDR and the (**b**) HDR images of the fluorescent dye phantom (Online Methods, Supplementary Figure 6) shown in Fig. 2, with the corresponding rHDR and HDR histograms (**c,d**). Region γ, light green. Region δ, green. Region ε, dark green. The HDR image is stored with 64 bits. The remapped image (<16 bits) facilitates visualization and allows for qualitative discrimination between the different areas of the image. The rHDR image is needed to display the 64 bit HDR data in print or bit-depth limited digital displays. For instance, note that the difficulty in discriminating between region γ and the background β in the HDR image even though mathematically the two regions are well resolved. The two regions however are visually well separated in the rHDR image. Image color bar: blu, dark noise signal; red, saturation levels. Scale bar, 50 μm.

Supplementary Figure 8

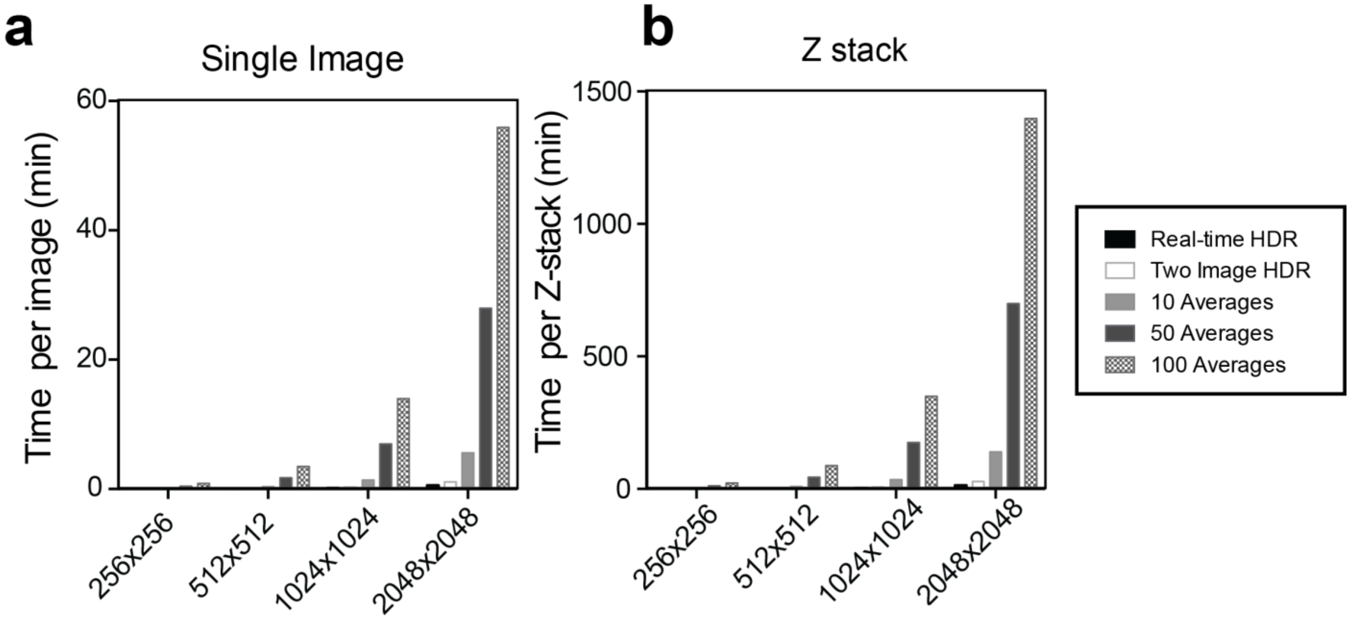

Comparison of acquisition times for different imaging acquisition schemes.
(**a,b**) Comparison of acquisition times between averaging and HDR imaging, acquired using sequential and simultaneous acquisition schemes. In addition to improved SNR, the HDR method is advantageous for accelerated acquisition of large datasets, which require signal averaging, in particularly when acquiring 3D imaging stacks.

Supplementary Figure 9

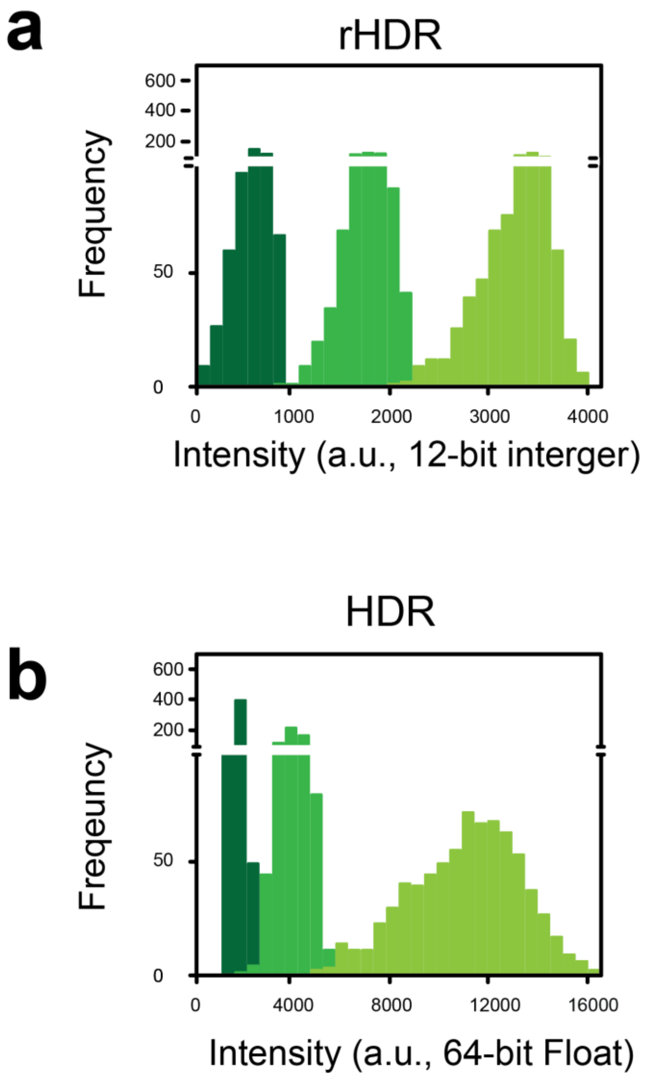

Comparison between HDR and remapped HDR histograms.
Comparison between the rHDR (**a**) and HDR (**b**) histograms obtained by imaging the bead sample presented in Fig. 3. While the information is the same between both HDR and rHDR images, it is not possible to visualize the three beads populations in the 64-bit HDR image as the display is limited to less than 16-bits. Remapping allows for a clear separation between the three populations of beads as observed in the remapped histogram and the corresponding rHDR image. Bead population 3, light green. Bead population 2, green. Bead population 1, dark green.

Supplementary Figure 10

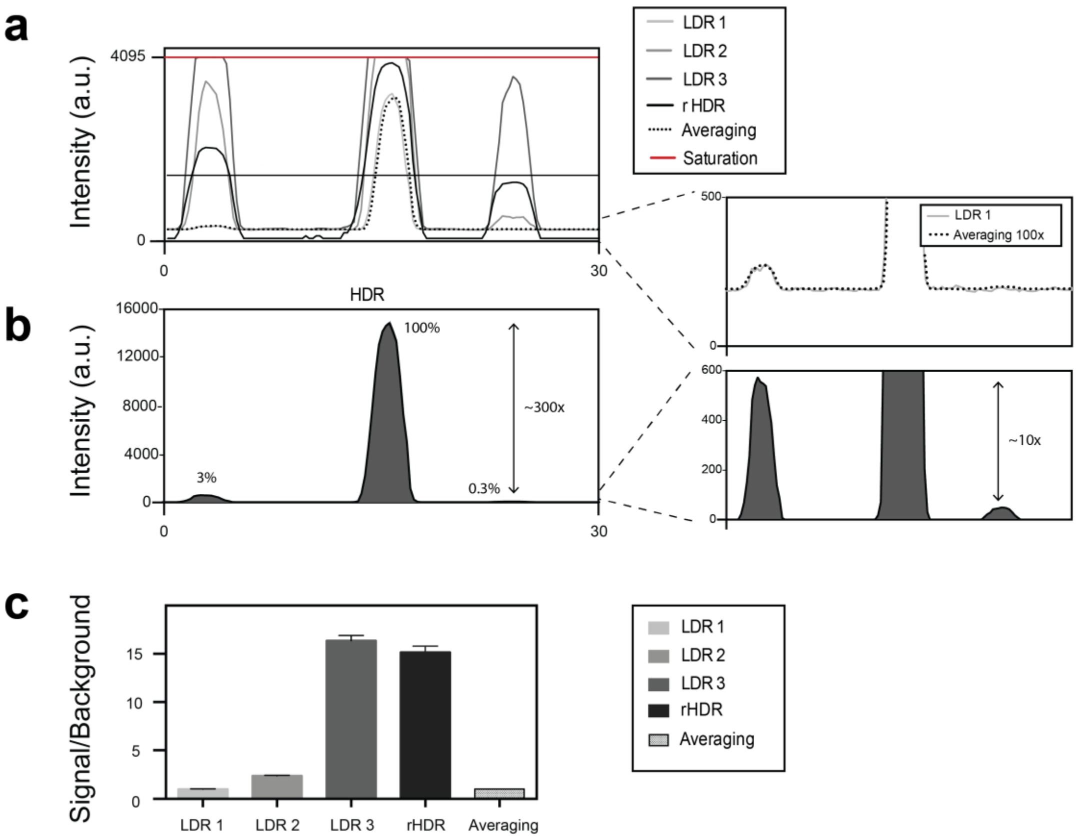

Bead profiles and Signal-to-Background.
(**a**) Intensity profiles obtained from bead images along the line indicated in Fig. 3a, for the three acquired LDR images, a 100 times averaged LDR image, and the corresponding rHDR image. Here the line is crossing along three beads with different fluorophore concentrations (3%, 100%, and 0.3% respectively). (**b**) Intensity profile of the HDR image. The HDR profile corresponds to the correct factor of approximately 300x and 33x between the 100% and 3% beads and between the 100% and 0.3% beads, respectively (Online Methods). Due to the large signal dynamic range of the sample and the limited detector dynamic range, the profile of the low concentration bead is buried within the noise in the LDR image and the averaged image, but it is clearly distinguishable in the HDR profile (**b**) and the remapped HDR profile (**a**). Increased SNR is present in the rHDR images (**c**).

Supplementary Figure 11

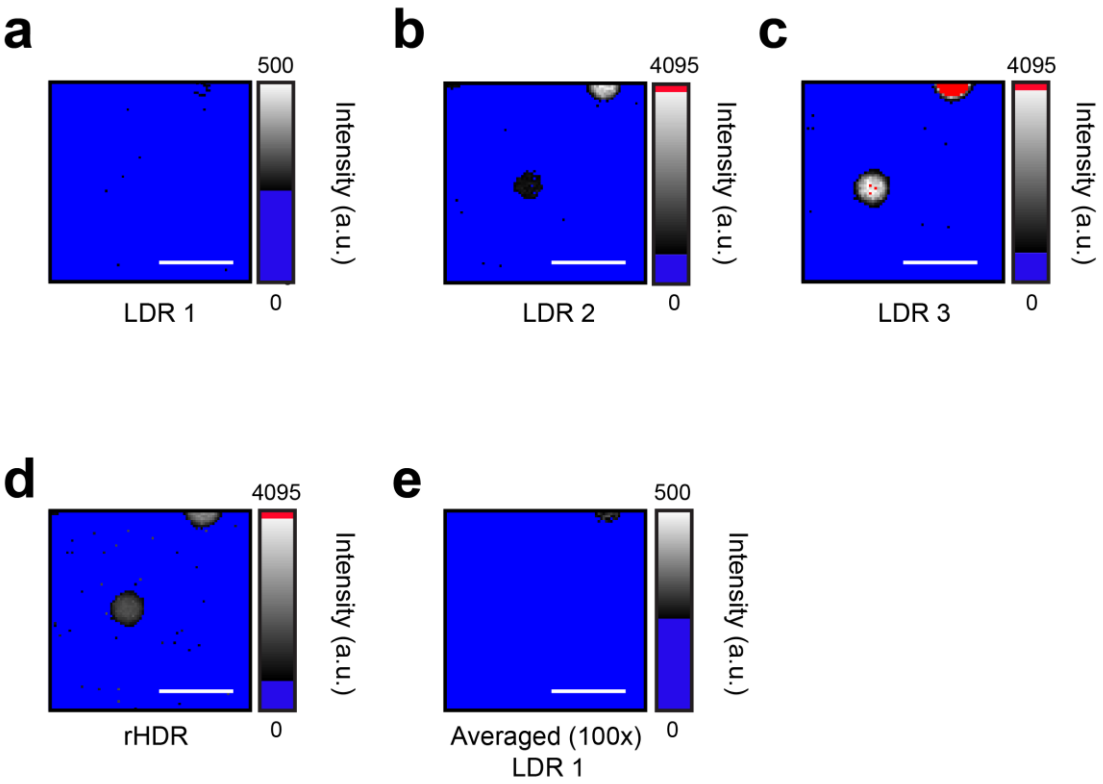

Comparison between two-photon averaging and HDR imaging.
(**a**,**b**,**c**) LDR images of the low concentration bead indicated in the boxed area of Fig. 3g. The bead is completely buried below the dark noise in (a). (**d**) rHDR image. (**e**) Averaged image obtained by averaging 100 acquisitions obtained under the same imaging conditions (no bleaching was observed). Despite averaging, only the HDR and rHDR images capture the large range of fluorescence signal present within the sample and show the structural information relative to the low concentration bead. Image color bar: blu, dark noise signal; red, saturation levels. Scale bar, 5 μm.

Supplementary Figure 12

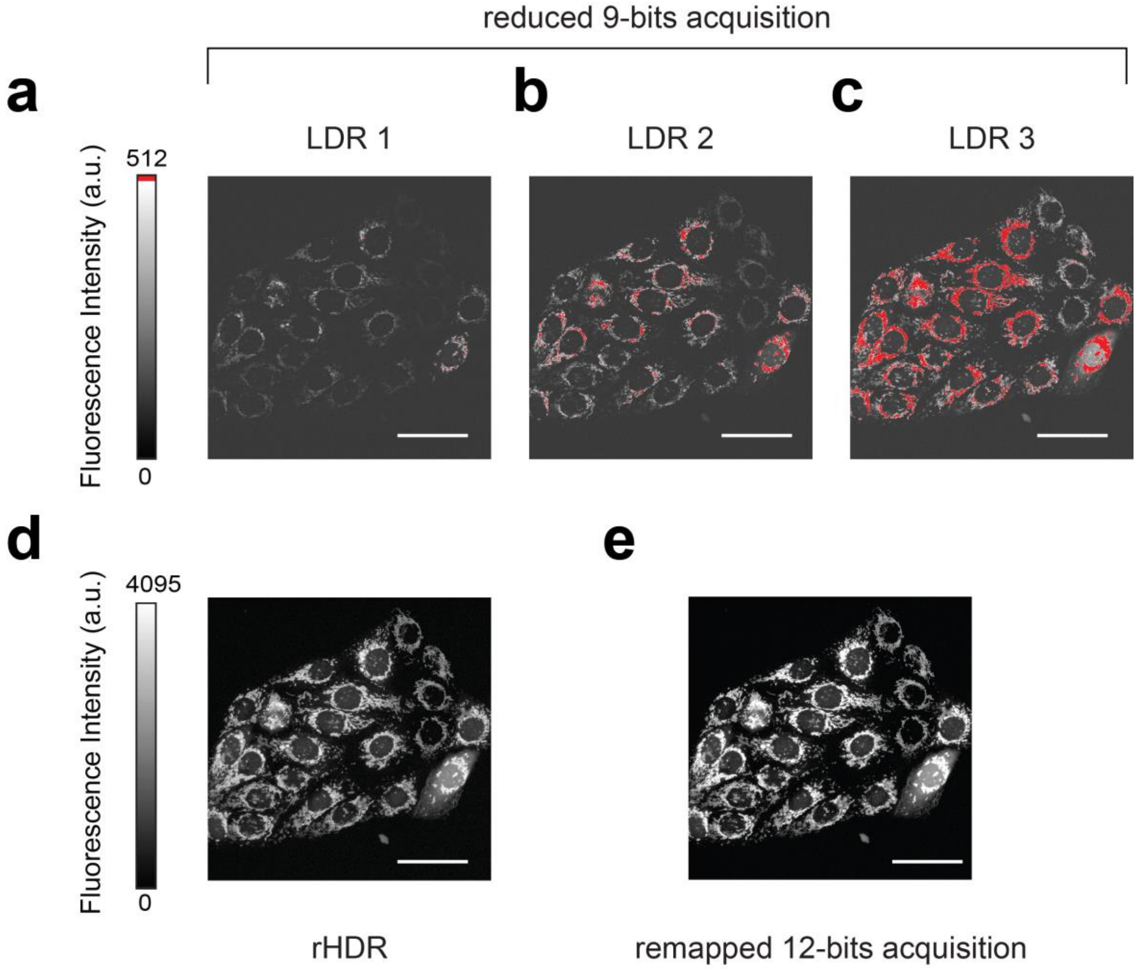

HDR reconstruction accuracy

The accuracy of HDR reconstructed images is tested by performing HDR acquisitions using a reduced bit depth (9 bits) of the PMT's dynamic range and comparing with an image of the same sample acquired using the full 12 bit depth (full dynamic range) of the PMT's, herein considered the ground truth. (**a-c**) 9 bits depth LDR images of OVCA-429 cells transduced with a GFP reporter of mitochondria. (**d**) The rHDR image obtained from the 9-bit LDR images acquired in (a-c). (**e**) A remapped image of the same sample acquired using 12-bits (the full dynamic range of the PMT). A comparison between the rHDR image (d) and a remapped image of the same sample acquired with the PMT full dynamic range (e) demonstrates the accuracy of our reconstruction method. Image color bar: red, saturation levels. Scale bar, 50 μm.

Supplementary Figure 13

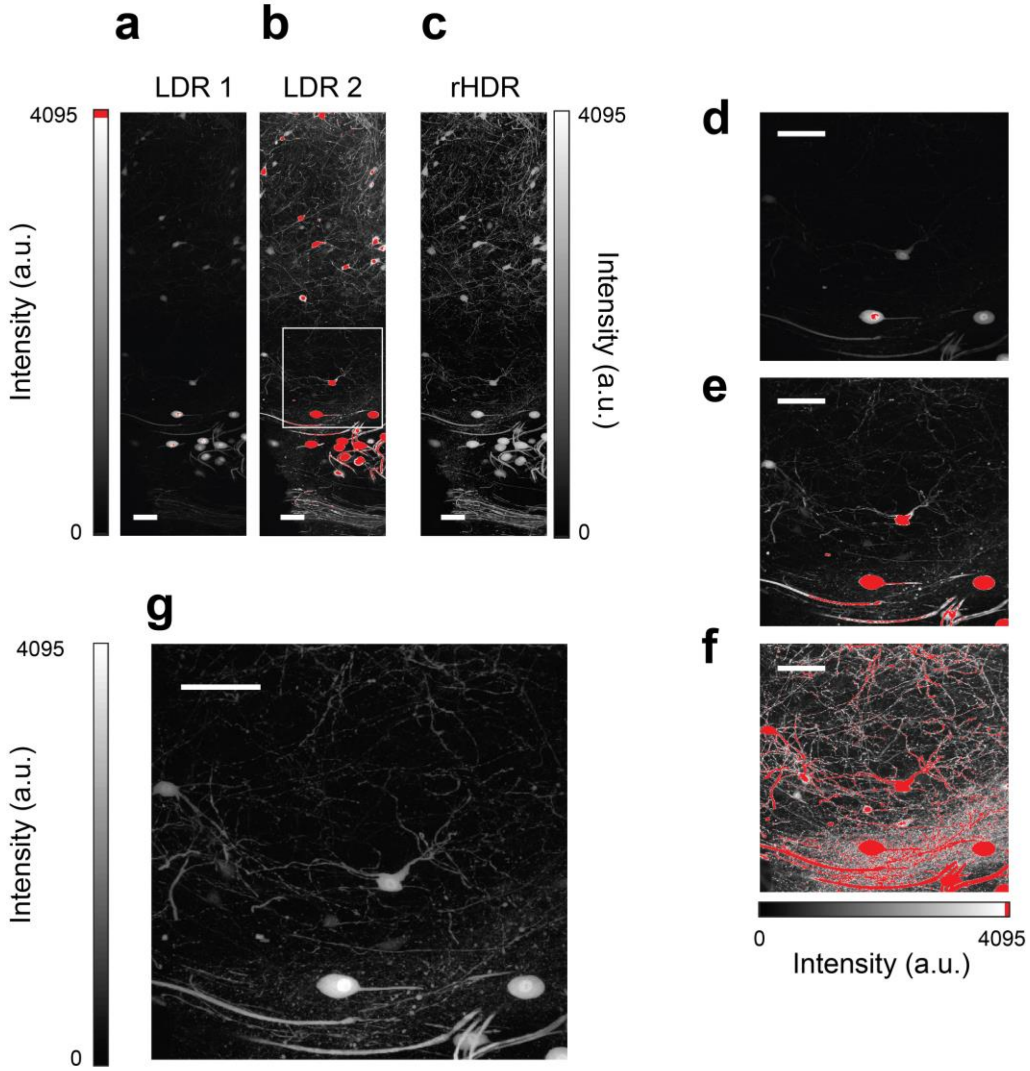

Whole brain imaging - 1
(**a,b**) LDR and (**c**) rHDR images of the boxed area of Fig. 5d. (**d-f**) Three different LDR images in an area with a large range of fluorescence signal. (**g**) Reconstructed rHDR image. Image color bar: red, saturation levels. Scale bars, 100 μm.

Supplementary Figure 14

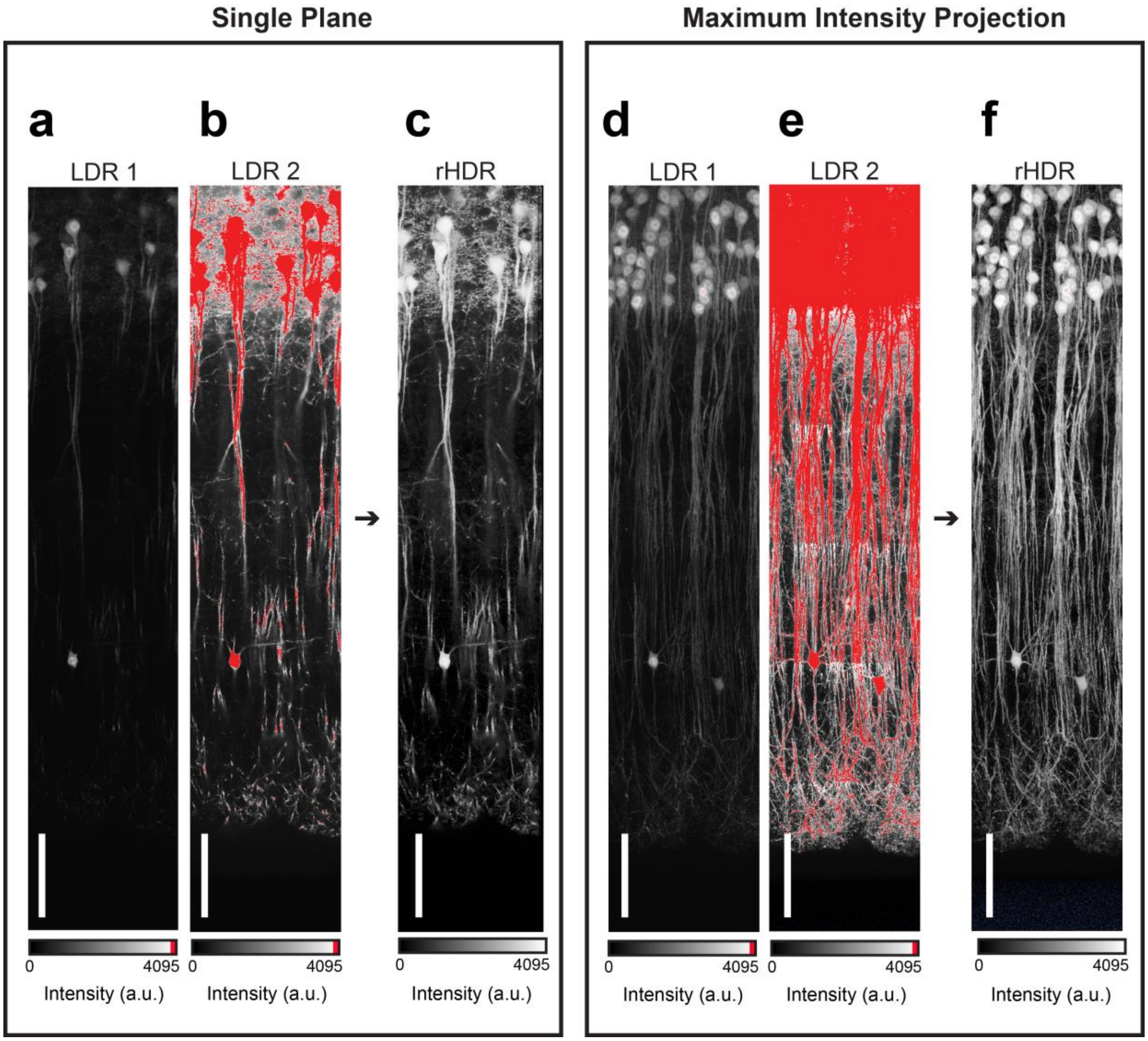

Single plane and maximum intensity projection HDR imaging
Two-photon HDR imaging of pyramidal neurons projecting into the cerebral cortex. Comparison between (**a-c**) single plane and (**d-f**) maximum intensity projections (MIP). For both cases the LDR (a,b,d,e) and rHDR (c,f) images are shown. Two-photon HDR imaging is particularly effective for MIP representations due to the large fluorescence range present within the volume of the cortex. Image color bar: red saturation levels. Scale bar, 200 μm.

Supplementary Figure 15

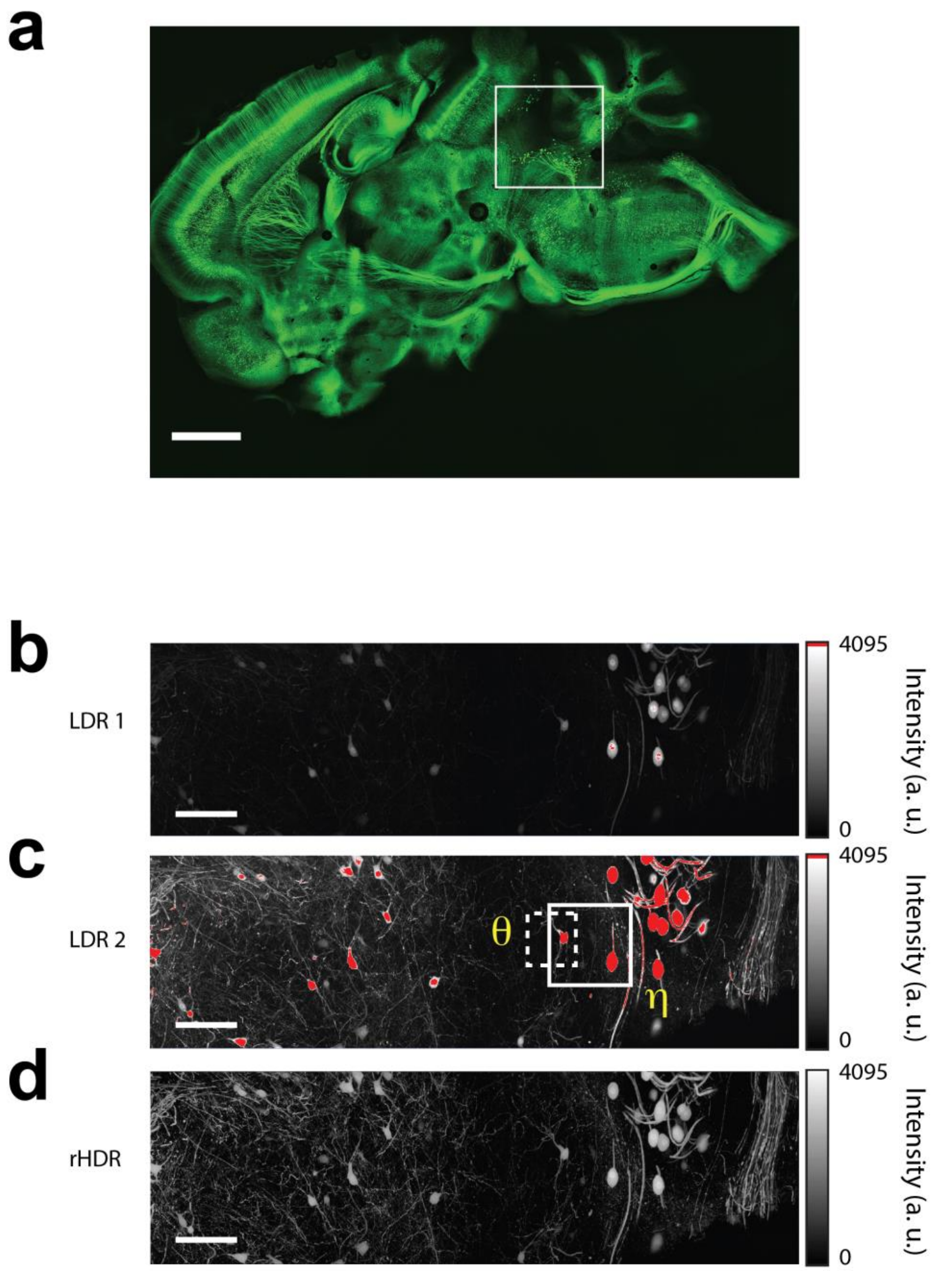

Whole brain imaging - 2
A representative rHDR image of a whole brain section (**a**) and magnified area (**b-d**). The rHDR image (d) emphasizes the very large fluorescence signal range present within this region of the brain. The boxed areas θ and η in (c), refer to the volumes used for 3D segmentation in Fig. 6 g-i. Image color bar: red, saturation levels. (a) Scale bar, 250 μm. (b) Scale bar, 150 μm.

Supplementary Figure 16

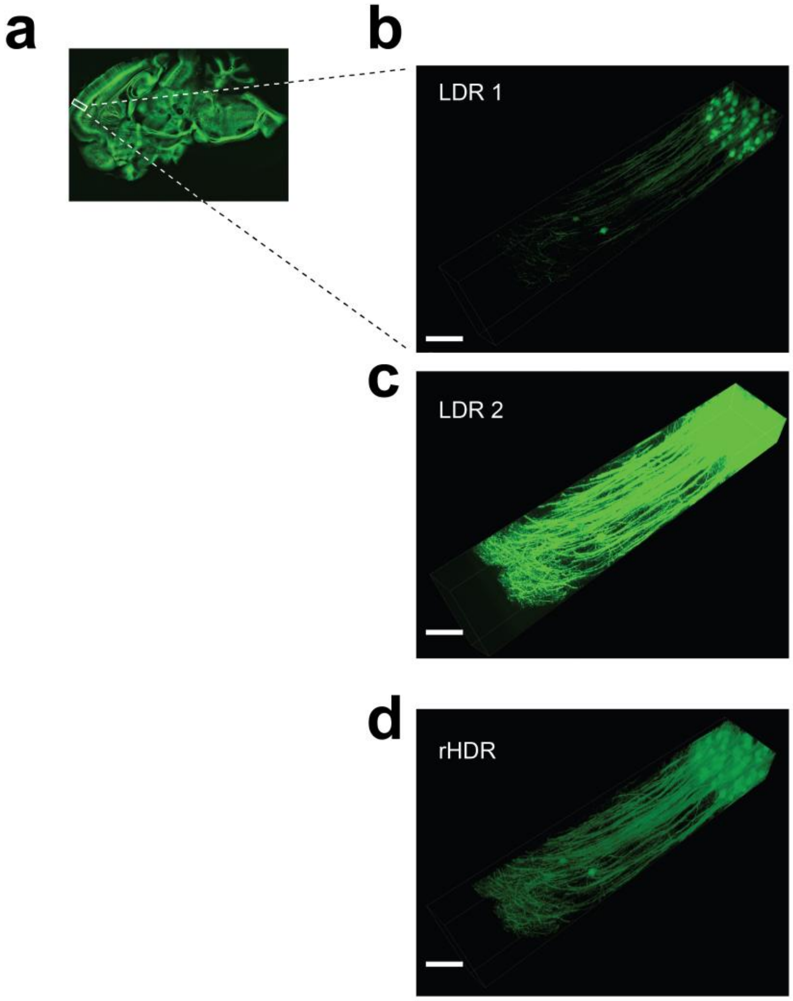

3D two-photon HDR imaging of pyramidal neurons.
Two-photon HDR imaging (**a**) can be extended to multiple planes for high-resolution 3D reconstructions. Both LDR (**b,c**) and rHDR (**d**) 3D volumes are shown. The large range of fluorescence signal present across the entire volume emphasizes the necessity of this method for accurate 3D segmentation. The non-saturated low dynamic range volume lacks information near the cerebral cortex. The saturated low dynamic range volume contains information with increased SNR proximal to the cortex, while the distal saturated area is not visible. The HDR volume, however, contains information about both regions and can thus be used for accurate automatic segmentation. Scale bar, 100 μm.

Supplementary Figure 17

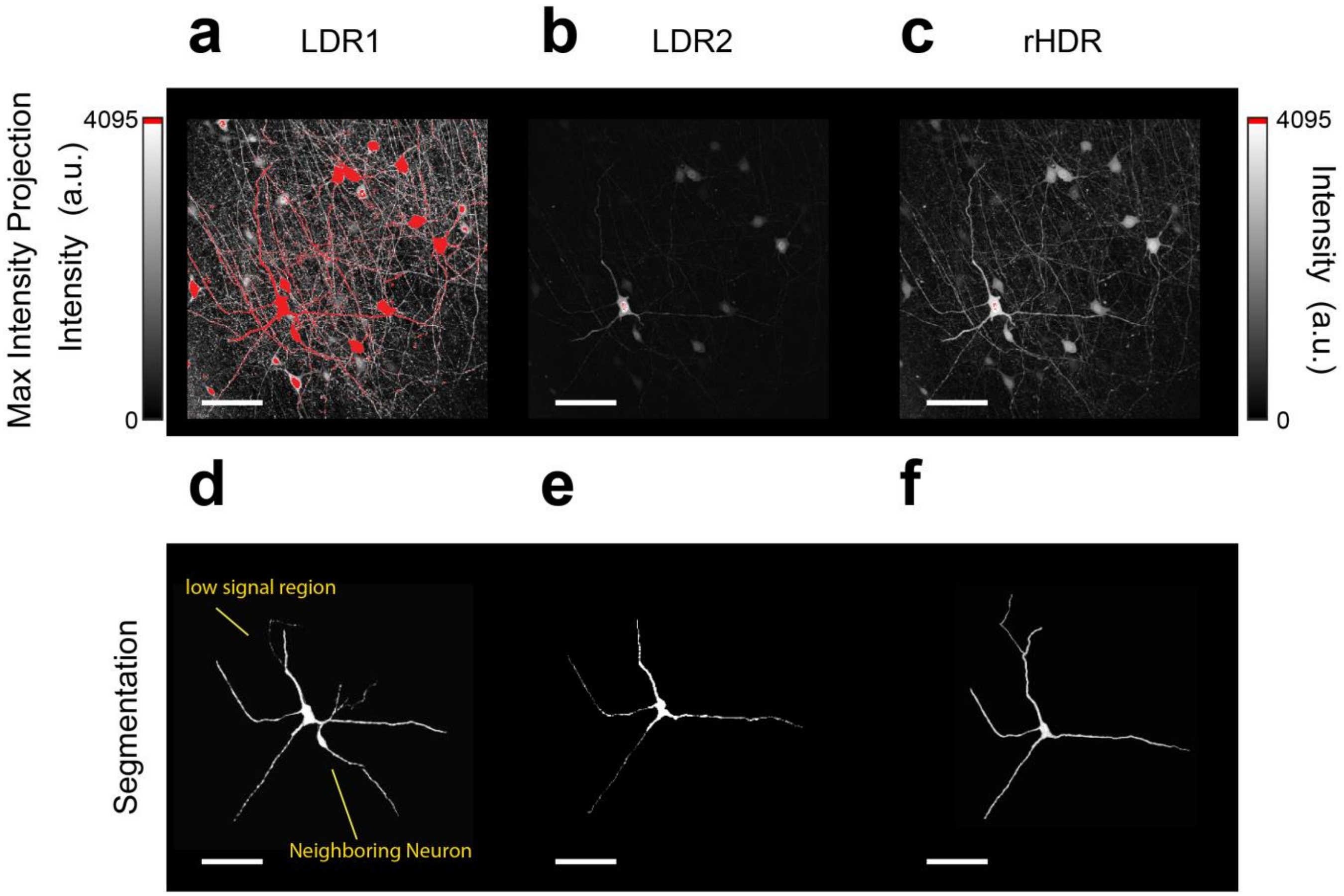

Single cell segmentation

(a-c) Maximum intensity projections of both LDR and associate rHDR images of the area indicated in Fig. 5d. **(d-f)** Supervised automatic 3D segmentation of a single cell obtained from both LDR and HDR datasets. Structures such as multiple cells and adjacent cell bodies in saturated signal regions in the LDR volumes tend to “fuse” together. In low signal regions, in LDR volumes, neuron processes tend to be fragmented. Proper segmentation can be automatically achieved on the 3D HDR volume as can be verified under visual inspection. Image color bar: red, saturation levels. Scale bars, 100 μm.

Supplementary Figure 18

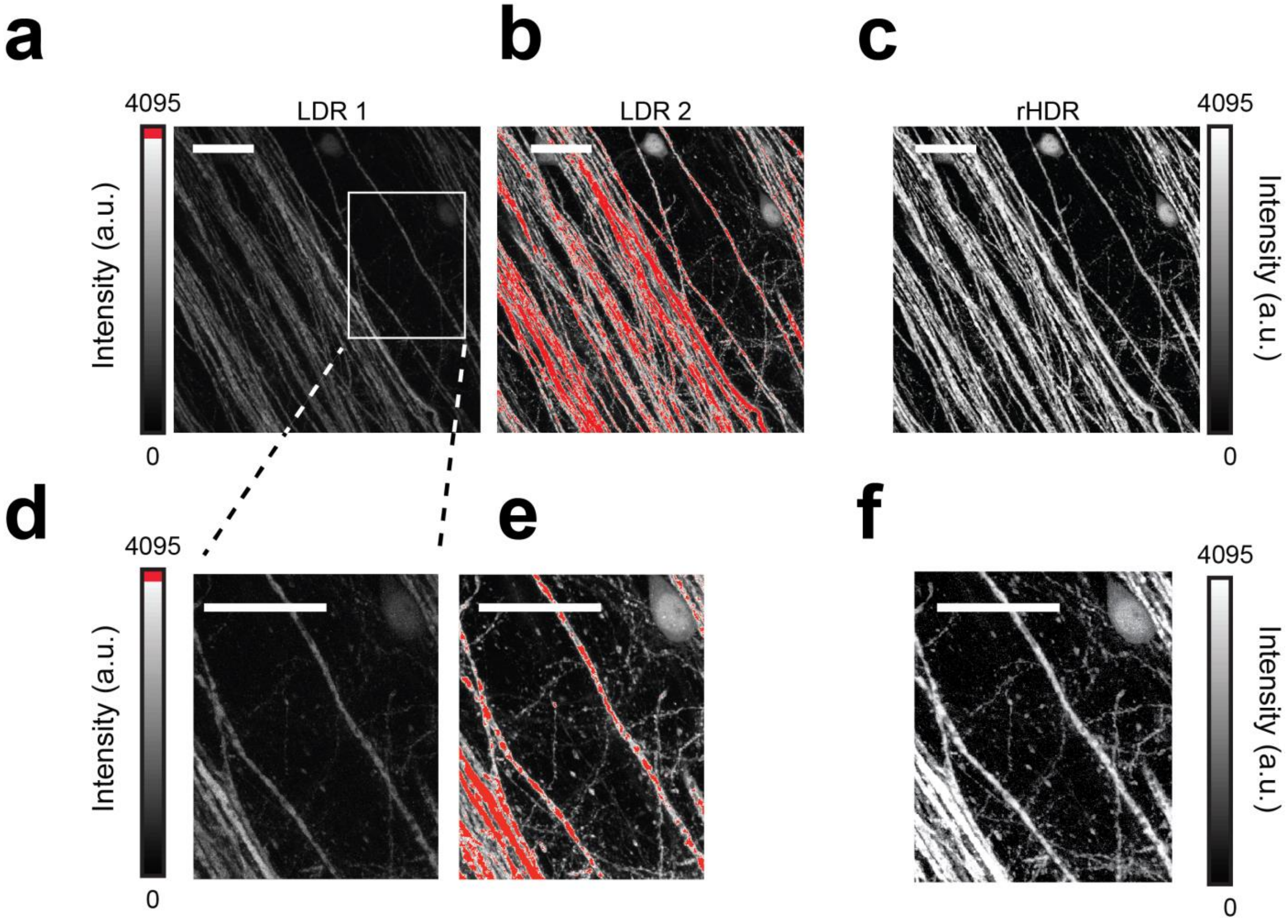

Imaging of neural spines.
(**a,b**) LDR and (**c**) rHDR images of the boxed area within Fig. 5d. (**d-f**) Zoomed in areas. The improved SNR and contrast in the HDR and the rHDR image, reveals neural spine structures otherwise not present in the low dynamic range image, as they are below the background noise. Image color bar: red, saturation levels. Scale bars, 20 μm.

Supplementary Figure 19

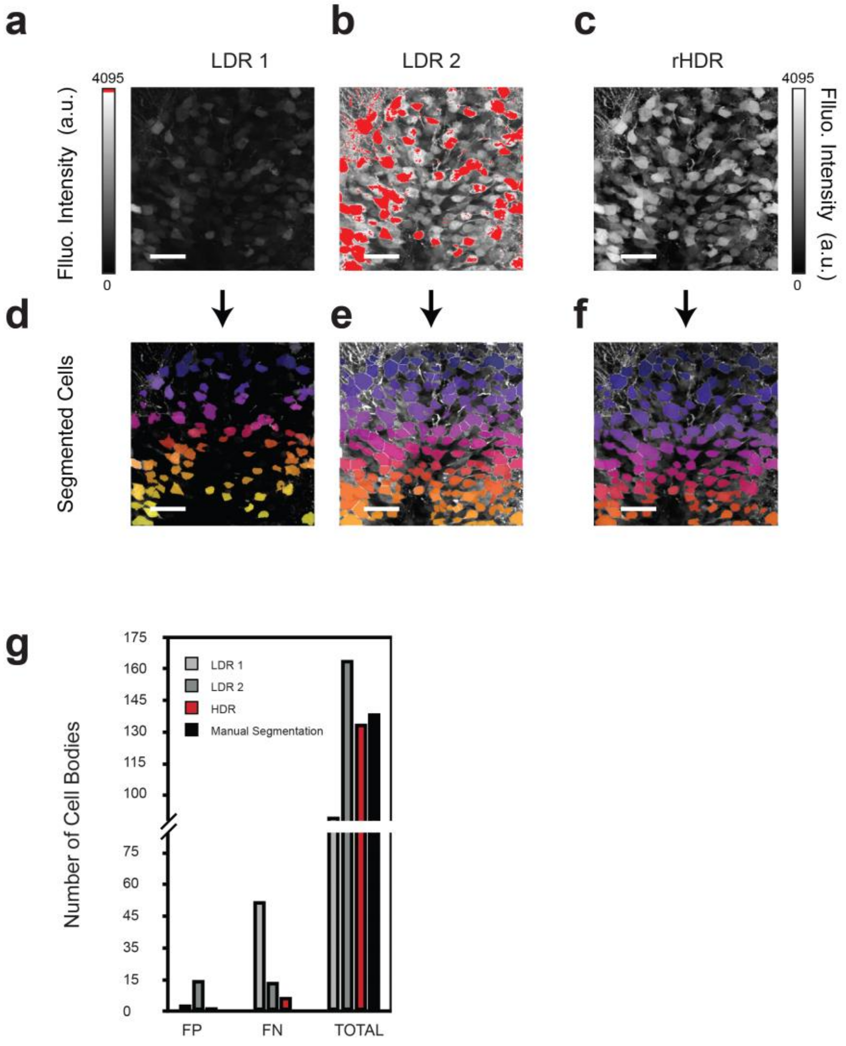

Cell body segmentation (densely populated area).

HDR imaging allows for accurate quantification of cell bodies in densely populated fixed tissue specimens. Here an area proximal to the hippocampus is selected. (**a,b**) LDR and (**c**) rHDR images. Segmentation and labelling of cell bodies for (**d,e**) LDRs and (**f**) HDR images obtained using a trainable Weka segmentation algorithm (see Online Methods). Colors are used to help to visualize and distinguish among the different cell bodies present within the field of view. (**g**) To determine the improvement in performance of the segmentation approach across the different images, a direct comparison is made among automatic segmentation applied to both LDR and HDR images and manual segmentation (here used as reference). The barplots highlight the improved segmentation accuracy of HDR images compared to LDR image of the same region. Sensitivity and specificity of cell detection are computed based on the amount of false positives FP (miss classified regions), false negatives FN (missed cells) and total number of cell bodies. Image color bar: red, saturation levels. Scale bars, 100 μm.

Supplementary Figure 20

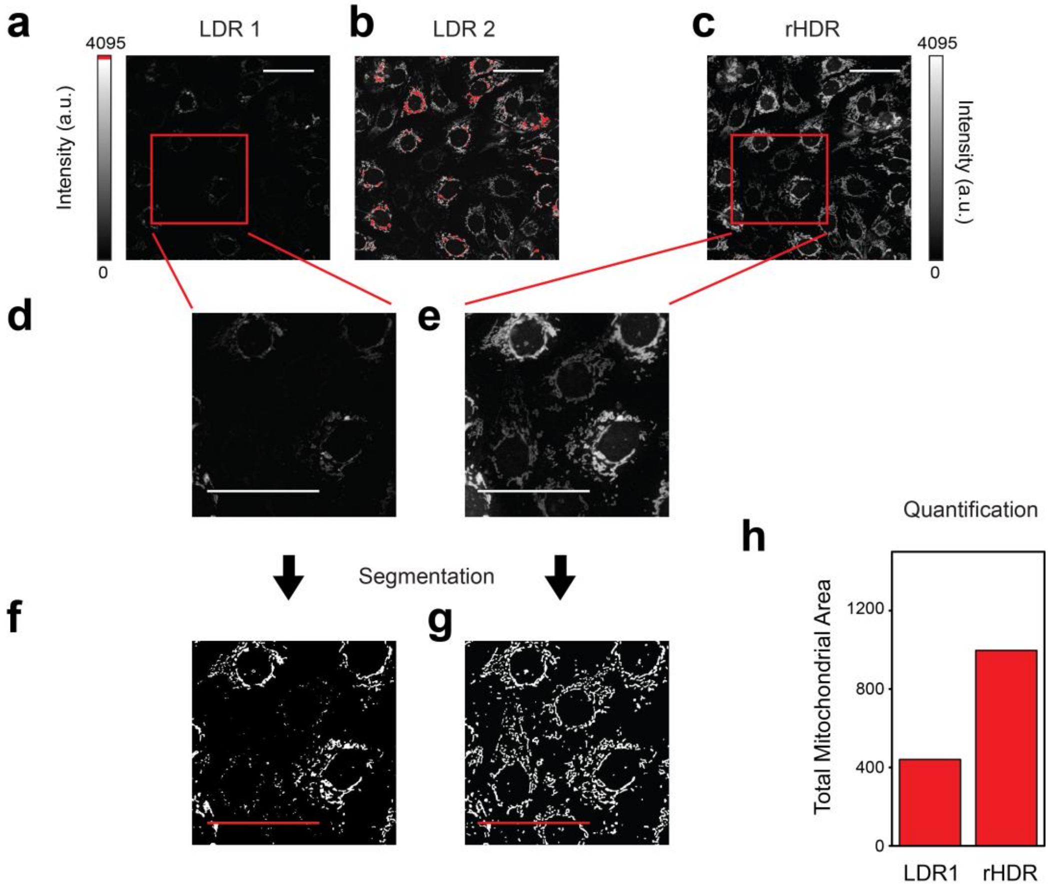

Mitochondrial segmentation

HDR imaging allows for accurate quantification of mitochondria in fixed cells. (**a,b**) LDR and (**c**) rHDR images of OVCA-429 cells transduced with a GFP reporter of mitochondria. A magnified (**d**) LDR and (**e**) rHDR image of the red boxed area in (a,c). (**f,g**) Mitochondrial segmentation of the LDR and rHDR images in (d,e). Due to the heterogeneous fluorescent expression found in the GFP genetic reporter of mitochondria, the rHDR image contains structural information that is not observed in the LDR image, where cells with low fluorescence intensity signal are buried within the noise level or present very low SNR. Here the segmentation on both images was performed using previously developed algorithms (Online Methods). (**h**) Total mitochondrial area as segmented from LDR and rHDR images. The increase of information content present in the rHDR image, results in improved identification and segmentation of mitochondrial bodies. Image color bar: red, saturation levels. Scale bar, 50 μm.

Supplementary Figure 21

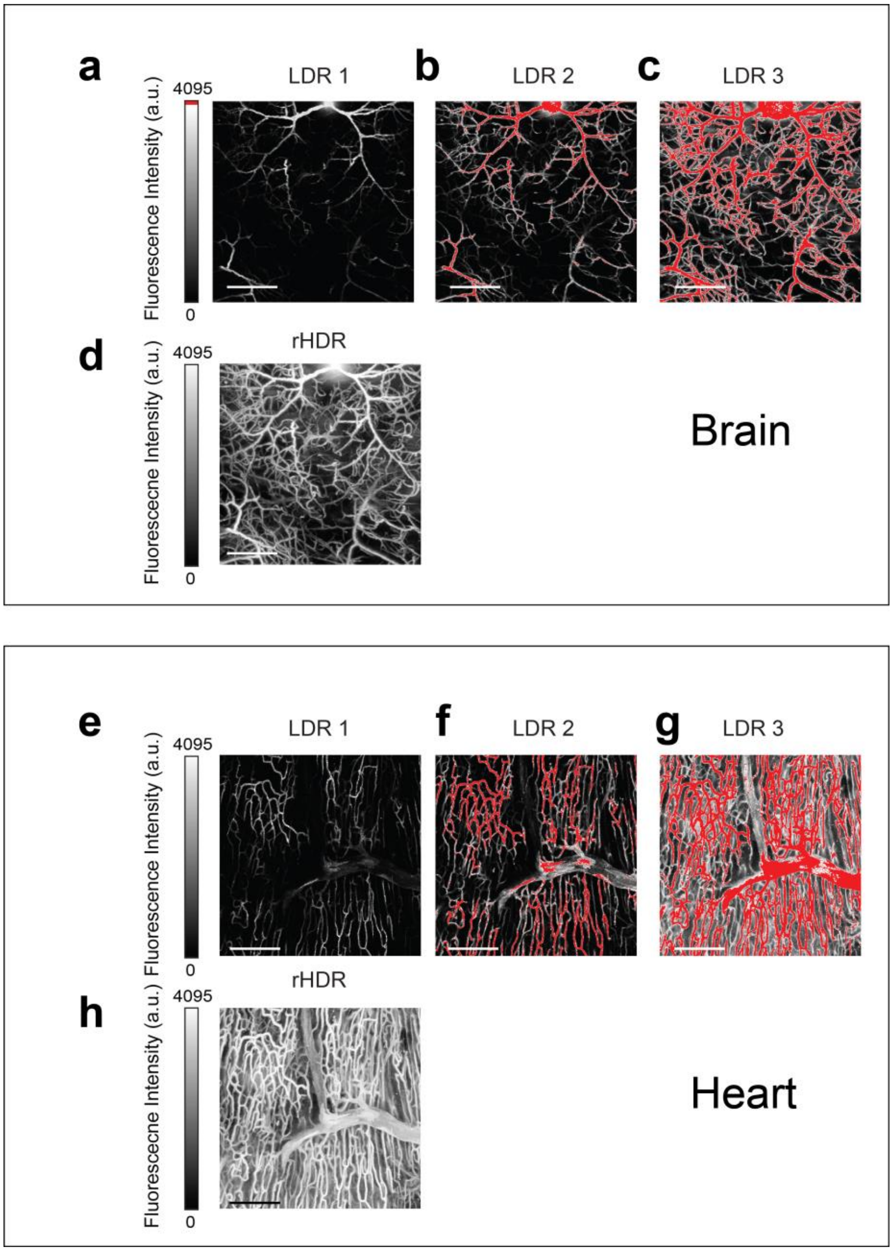

HDR vasculature imaging in cleared organs.
HDR imaging of the brain and heart vasculature. Brain (**a-c**) LDR and (**d**) rHDR images. Heart (**e-g**) LDR and (**h**) rHDR images. The stained vasculature of different organs present substantial differences in fluorescence signal due to variations in dye labeling, dye uptake, or differences in blood vessels size. A large increase in SNR and information content is present in the reconstructed HDR images. Vasculature is labeled with DiI (Online Methods) and organs are cleared (Online Methods). Image color bar: red, saturation levels. Scale bar, 125 μm.

Supplementary Figure 22

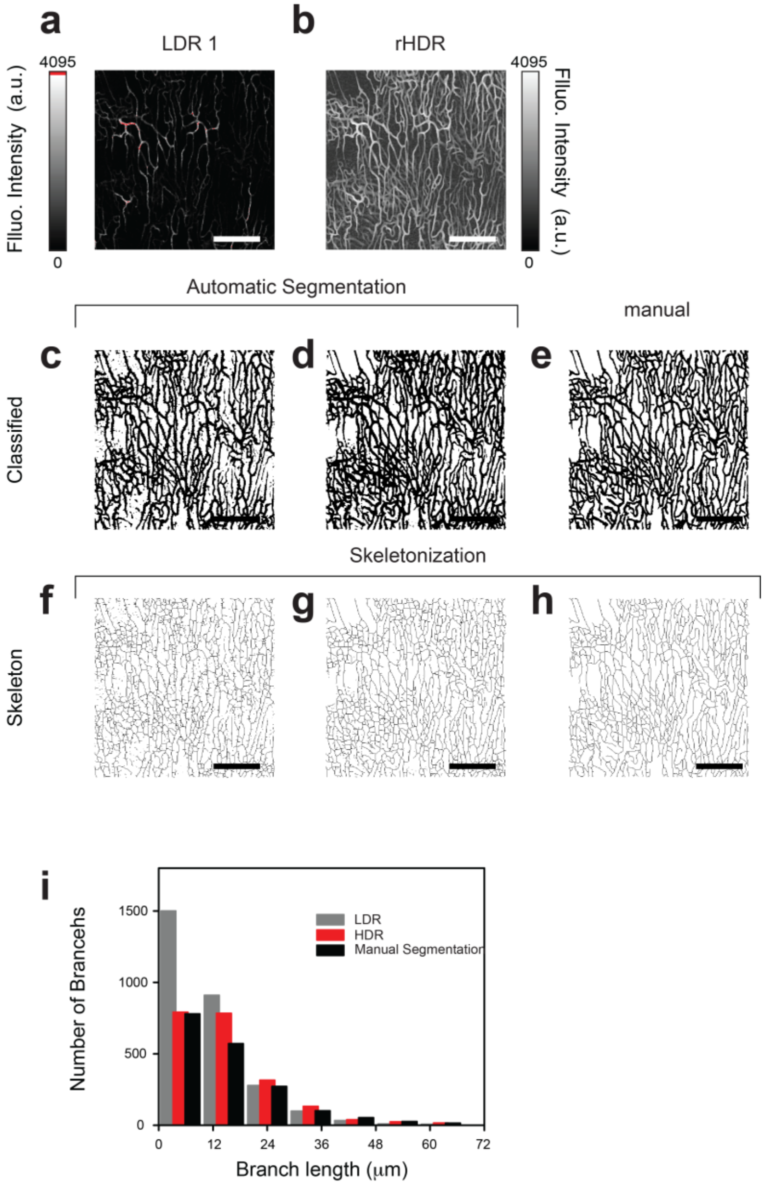

Vasculature segmentation.

Vasculature segmentation of a cleared DiI stained heart (Fig. 9). Segmentation is performed on both low LDR1 (**a**) and remapped HDR (**b**) images. (**c,d**) Segmentation of the vasculature network on LDR and rHDR images (Online Methods). (**e**) Classification is also done manually on the rHDR image and used to establish the ground truth. Skeletonization of segmented LDR1 (**f**), rHDR (**g**) and manually segmented (**h**) images respectively. (**i**) Barplot of the microvascular morphology extracted from the skeleton images. An excellent agreement of the number of branches for a given branch length was observed between manual and automatic segmented HDR images. Conversely, LDR image quantification resulted in overestimating the number of short branches, inducing a high degree of vascular fragmentation, likely due to poor SNR. Image color bar: red, saturation levels. Scale bar, 150 μm

Supplementary Figure 23

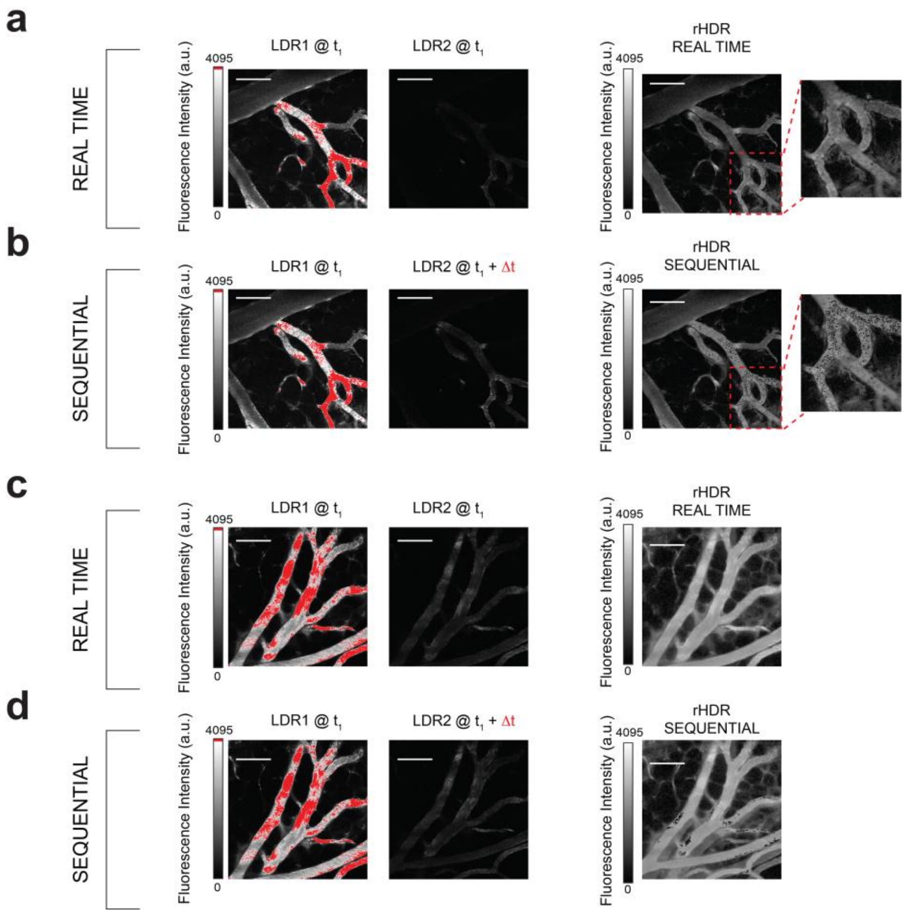

Absence of reconstructions artifacts during *in vivo* real-time HDR acquisitions.
The real-time simultaneous HDR acquisition method presented here demonstrates the ability to eliminate all HDR reconstruction artifacts present during sequential acquisition. This is crucial for *in vivo* imaging, where cardiac and respiratory cycles compromise the ultimate spatial and temporal imaging resolution required to robustly detect dynamic events occurring on a time scale faster than the acquisition time. (**a**,**b**) LDR and HDR *in vivo* images collected in real-time (a) and sequentially (b) in a dorsal window chamber implanted in a mouse. A bolus of 2MDa FITC-Dextran was injected intravenously and the vascular kinetics imaged (Online Methods). The rapid vascular kinetics of the tracer results in different intensity levels in sequentially acquired LDR frames, giving rise to strong artifacts during HDR reconstructions (right inset in b). These artifacts are absent in the reconstructed images from real-time acquisition (right inset in a). (**c**,**d**) Cardiac and respiratory physiology causes motion-induced artifacts during image acquisition. Different areas of sequentially captured images (d) may be misaligned in consecutive frames giving rise to severe ghosting artifacts in the final reconstructions. Motion artifacts are suppressed using our real-time HDR acquisition method due to the ability to simultaneously acquire light across the PMTs for each pixels. Image color bar: red, saturation levels. Scale bar, 125 μm.

Supplementary Figure 24

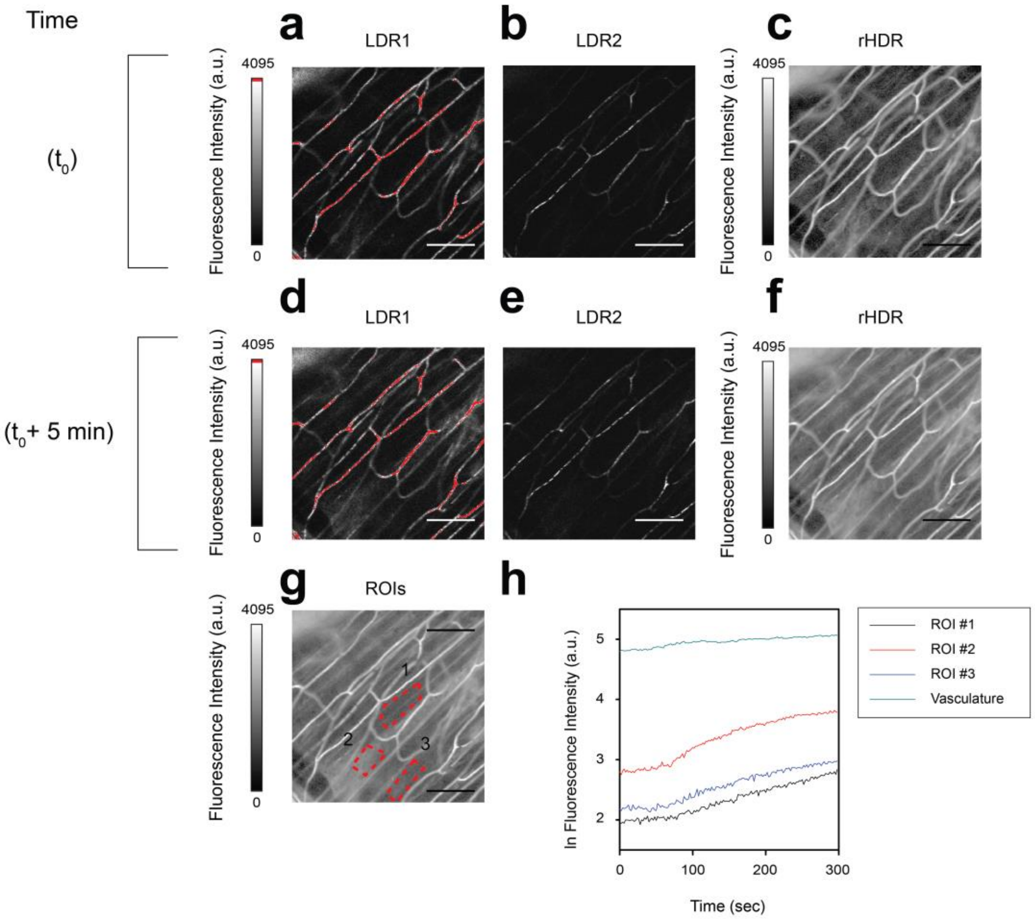

***In vivo* kinetic of vascular extravasation across different tissue compartments.**
The HDR imaging method presented here can be used for the *in vivo* real-time assessment of the time-intensity variations of imaging probe kinetics across multiple tissue compartments. LDR temporal sequences have a limited ability to capture the full dynamic range of fluorescence intensities during the kinetics of an intra-vascular 2MDa FITC-Dextran and a freely diffusible 4kDa FITC-dextran. Conversely, HDR imaging was capable of acquiring the full dynamic range of both the 2MDa and 4kDa probes both in time and between the different tissue compartments. LDR and rHDR images are shown at time zero (**a-c**) and 5 minutes later (**d-f**), following 4kDa FITC-dextran injection. (**g**) Regions of interest are selected in the extravascular compartments (ROIs 1-3) and in the vasculature network. (**h**) TICs calculated as the mean of the signal within the ROI and the vasculature network, are plotted on a *ln* scale as a function of time for both LDR and HDR time sequences. The high SNR and extended dynamic range of the HDR TICs demonstrate the ability to robustly quantify the vascular and extravascular kinetics of fluorescent dyes. Image color bar: red, saturation levels. Scale bar, 125 μm.

Supplementary Figure 25

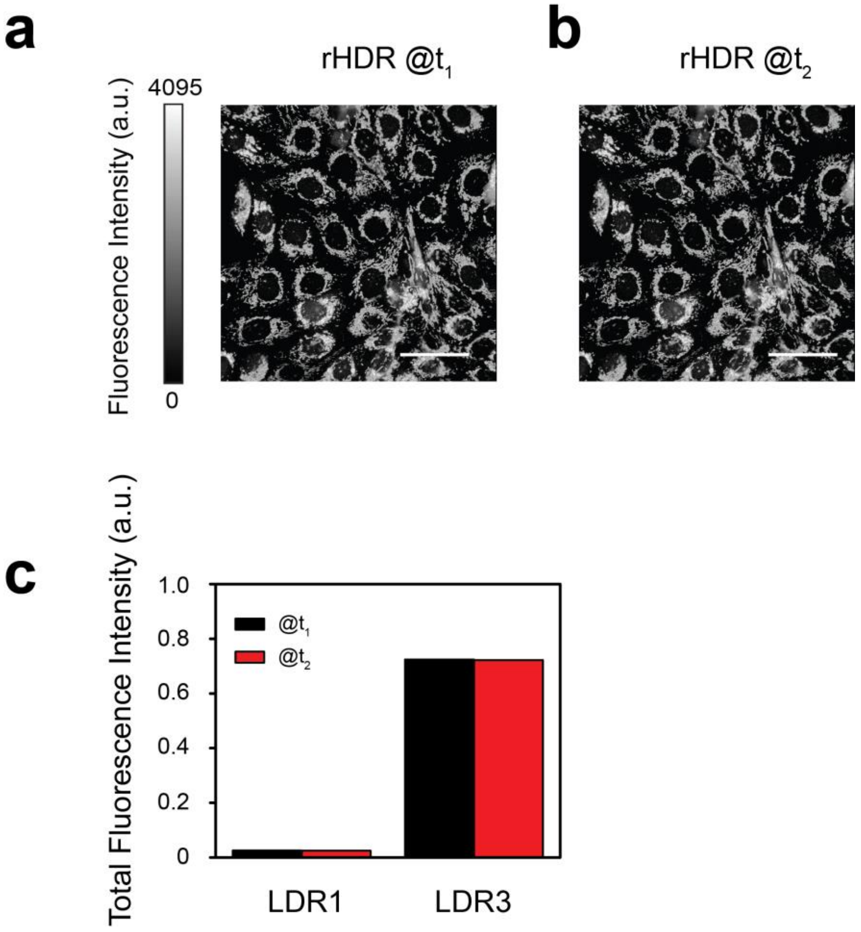

Lack of bleaching during HDR imaging acquisition.
No bleaching was observed during HDR imaging acquisition for all samples (cells expressing a GFP genetic reporter of mitochondria, cells stained with AlexaFluor-488 Phalloidin, DiI stained vasculature in both fixed and cleared tissue, brain tissue sections stained with Alexafluor-488 conjugated secondary antibodies). (**a**,**b**) rHDR images of the same cells and at the same optical imaging plane, acquired consecutively after each other. No difference or artifact due to photobleaching is present in the two images. (**c**) Total fluorescence intensity for the LDR images acquired at two consecutive times t_1,t_2. No significant difference in intensity is present in both LDR images. Image color bar: red, saturation levels. Scale bar, 50 μm.

Supplementary Figure 26

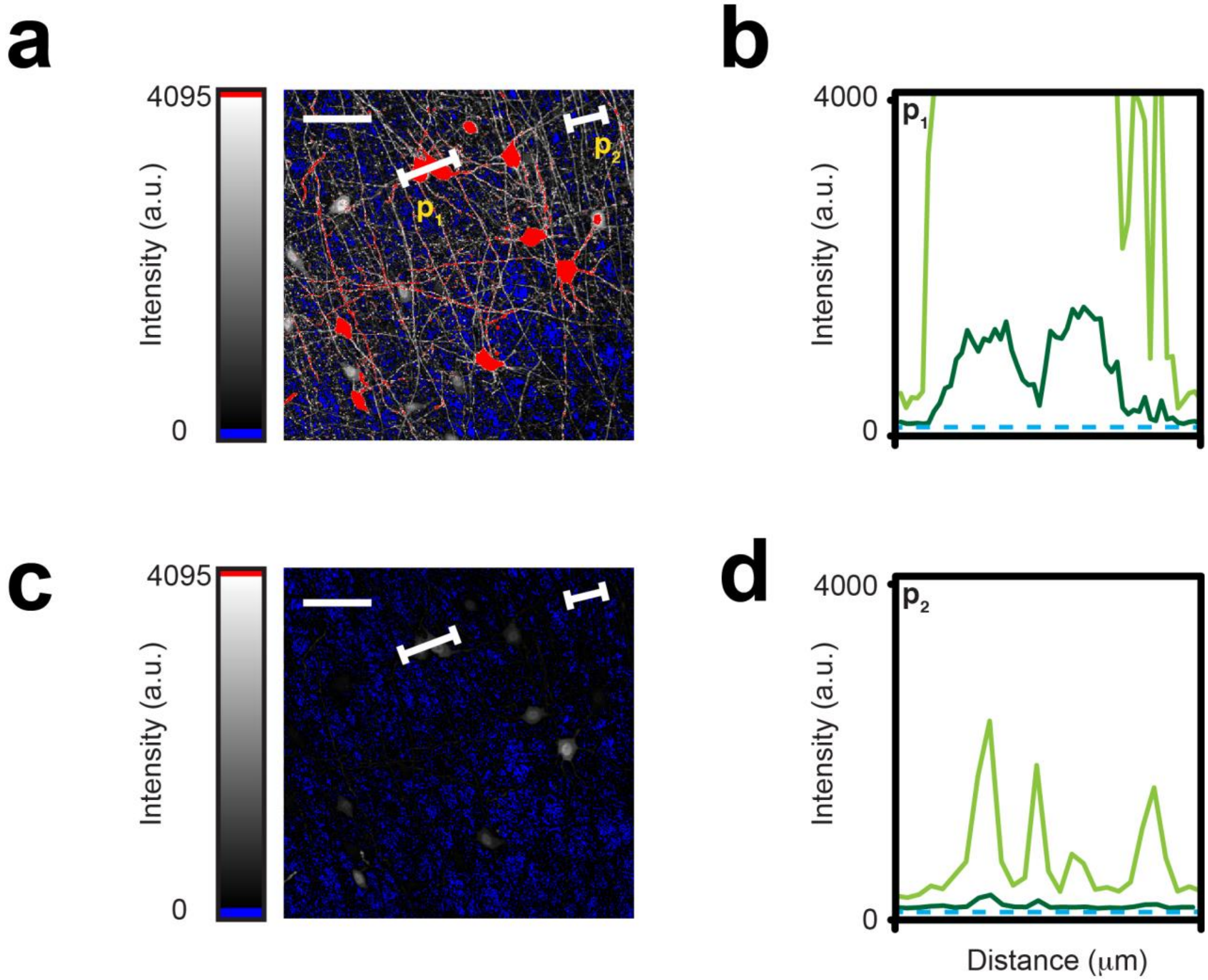

Large range of fluorescence signal prevents simultaneous display of structures with different intensities.

(**a**,**b**) A saturated low dynamic range (LDR) image of neural cells within the brain and intensity profiles along two different lines (white bars). Only the profile along the dim structures can be used to obtain structural information for proper quantification or segmentation. The profile along the saturated area provides no structural information (**c**,**d**). Non-saturated LDR image of the same area as (a) and the corresponding intensity profiles. Here the profile along an area of the image presenting low fluorescence signal is at the same level as the background noise (dark green) and cannot be used for visualization or segmentation. The same profile in the saturated image instead reveals the presence of fine cellular structures (light green). Image color bar: blue dark noise, red saturation levels. Scale bars, 100 μm.

Supplementary Figure 27

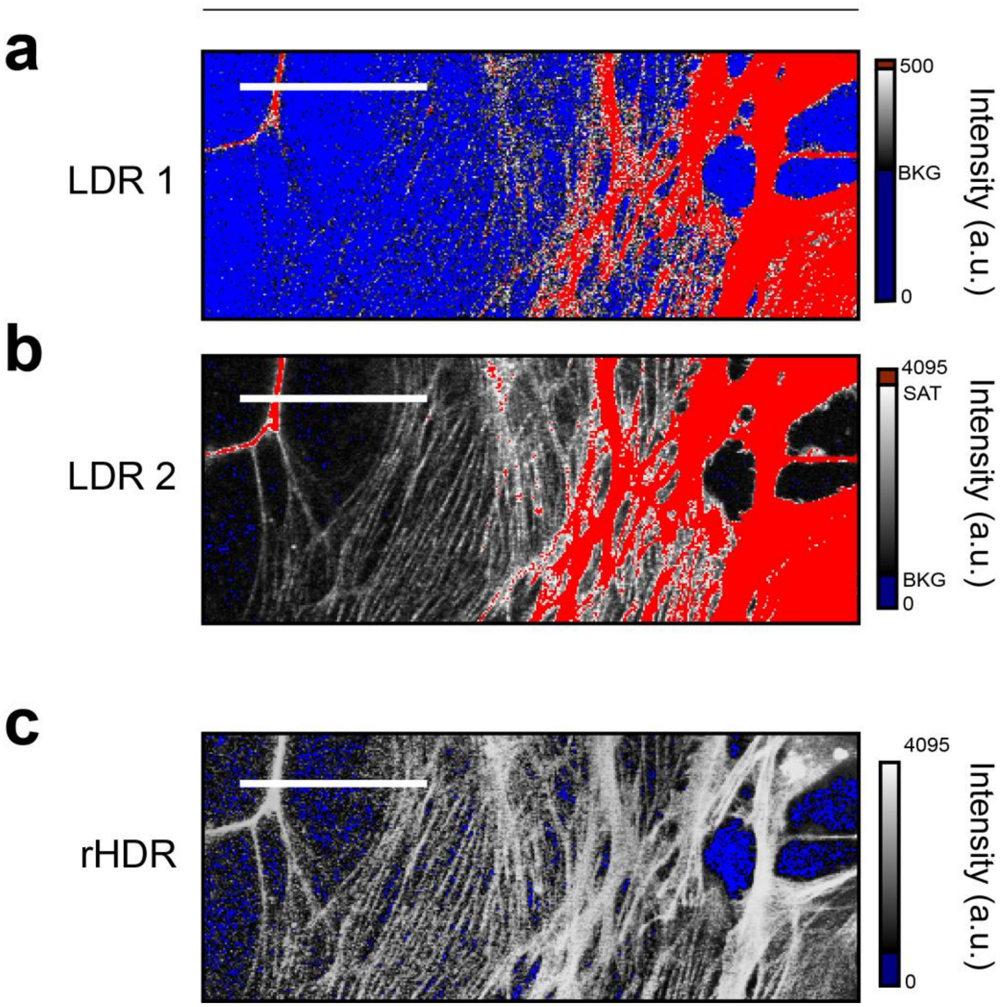

Extended range emphasizes the presence of cellular structural information.
(**a,b**) Low dynamic range and (**c**) remapped HDR images of the boxed area present in Fig. 4a of BS-C-1 cells. The unsaturated low dynamic range (LDR1) image is displayed on a reduced scale (0 to 500) to show the lack of structural information present in the low signal area (left region). The saturated low dynamic range (LDR2) image provides good SNR to differentiate between low intensity structures, but high intensity structures cannot be resolved. The remapped HDR image (rHDR) contains information from both the LDR images and emphasizes the presence of both high and low intensity structures. Image color bar: blue represents the dark noise, red saturation levels. Scale bar, 50 μm.

Supplementary Figure 28

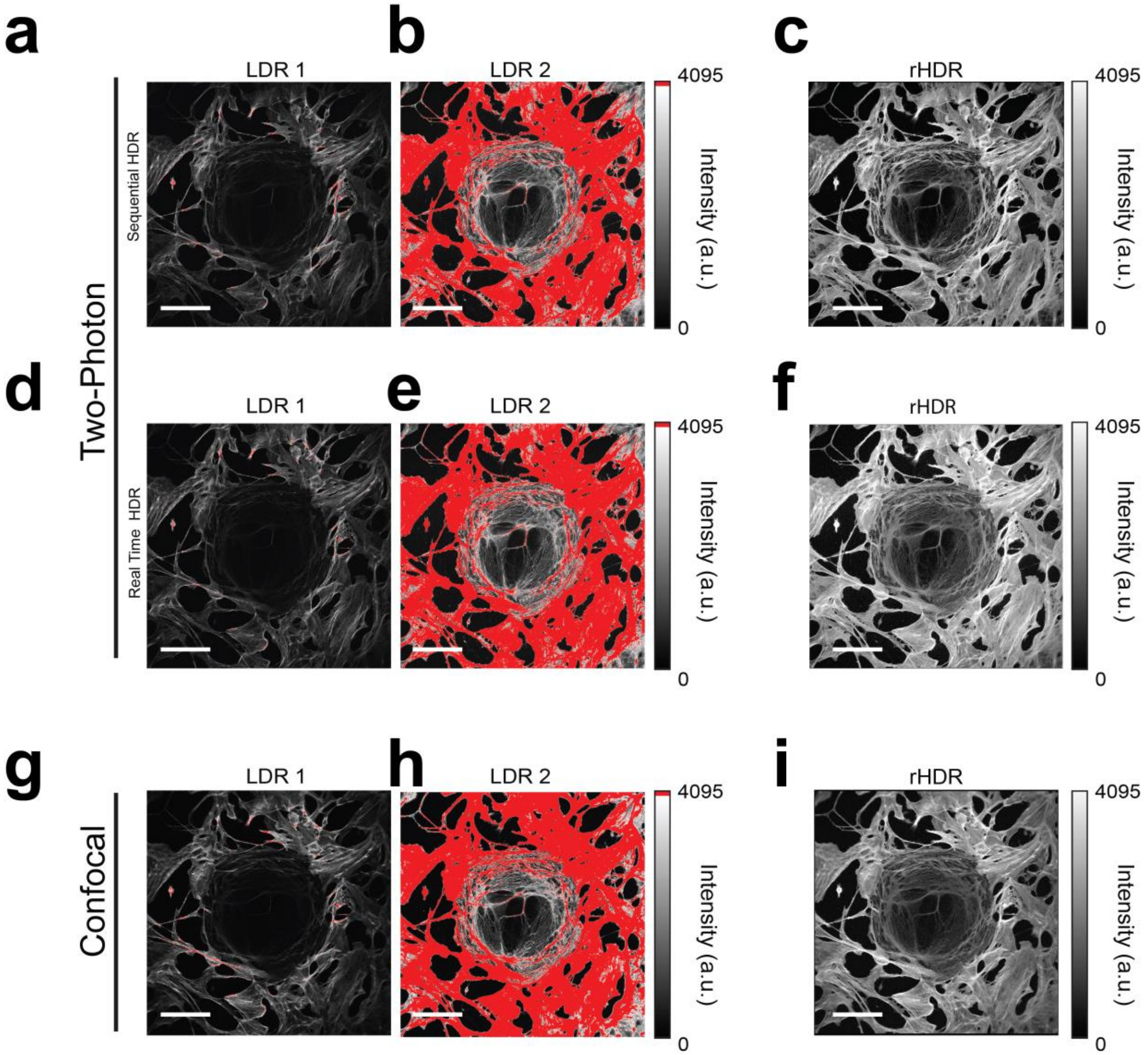

Two-photon and confocal HDR imaging comparison.

The imaging setup works for both two-photon and confocal microscopy. While two-photon imaging offers several advantages over one-photon excitation, not all probes can be excited when using standard Ti:sapphire laser, and confocal imaging is the only available option. Additionally, when imaging samples at low resolution with low numerical aperture objectives, such is the case for example for mice whole brain slices, two-photon excitation is not efficient and confocal imaging is more suitable for this approach. A comparison is shown between LDR and rHDR images of BS-C-1 cells stained for actin, acquired sequentially (**a-c**) or simultaneously in real-time (**d-f**) using two-photon or acquired sequentially in confocal microscopy (**g-i**). Image color bar: red, saturation levels. Scale bar, 100 μm.

Supplementary Note 1

High dynamic range image reconstruction. To create HDR images, the range of fluorescence is sampled with multiple (N) LDR images $\mathbf{I}_1$, $\mathbf{I}_2$, …$\mathbf{I}_\mathrm{j}$ …$\mathbf{I}_\mathrm{N}$. These images are then corrected for the response of the sensor and fused together to give rise to a final image $\mathbf{I}_\mathrm{HDR}$, which has a dynamic range greater than the ones of the individual LDR images. The HDR images are obtained using a pixel-wise fusion algorithm, such that the fusion rule explanation can be limited to a single HDR pixel, i, with generic coordinates (*x*,*y*). This approach is possible because the image acquisition is performed on a pixel-by-pixel basis for both confocal and multiphoton imaging modalities.

Specifically, each pixels value Z_{ij} of an LDR image $\mathbf{I}_j$, is mapped into the value T_{ij} by a transformation f. Both detector response r and modulator factor $\Delta\alpha_j$ (by varying $\Delta\alpha$, a different LDR image is taken) are known. The mapping is expressed by the following equation,

$$T_{ij} = f\left(r, Z_{ij}, \Delta\alpha_j\right) \tag{1}$$

The modulator parameter $\Delta\alpha_j$ sampling the jth subset of the PMT dynamic range can be obtained through a series of increasing power, integration times, or neutral density filters. In principle, these options should yield equivalent results. T_{i1}, T_{i2}, ... T_{iN} are obtained by changing the modulator value $\Delta\alpha_j$.

Then for a single pixel i, the HDR value is calculated as a weighed combination of T_{i1}, T_{i2}, ... T_{iN} values by the following equation:

$$I_{\mathrm{HDR},i} = \frac{\sum_{j=1}^{N} T_{ij} w(Z_{ij})}{\sum_{j=1}^{N} w(Z_{ij})} \tag{2}$$

Where w is a window function such that only Z_{ij} values above noise and below saturation are considered (Fig. 1a). $\mathbf{I}_{\mathrm{HDR}}$ is stored as a 64-bit floating-point number, whereas any original image $\mathbf{I}_j$,was digitized using a 12-bit integer.

Multiple images can be collected simultaneously with our custom-made two-photon HDR imaging system **(**Fig. 1b and Supplementary Fig. 2,4) and sequentially when operating in confocal mode (Supplementary Fig. 3) or alternatively in two-photon mode if only one single PMT is available. Simultaneous imaging allows for real-time collection of $\mathbf{I}_1, \mathbf{I}_2, \dots \mathbf{I}_j, \dots \mathbf{I}_N$ images, with negligible inter-frame movement. Therefore HDR images can be also obtained for non-stationary objects, live cells, and *in vivo.*

Supplementary Note 2

Dynamic range compression for visualization. Once HDR images are constructed, they can be directly processed for quantification and segmentation. Their visualization, however, is constrained to a reduced dynamic range by the display monitor. Thus, further processing is required in order to visualize the HDR image. Specifically, the bit-depth of the HDR image, $\mathbf{I}_{\mathrm{HDR}}$ which is stored as a 64-bit floating-point number must be reduced to less than 16-bit to be displayed. In this work, we remap to 12-bit unsigned integers for comparison with the original 12-bit raw images.

Simple global linear scaling will result in linearly flattened images and cannot be used to visualize the HDR image. Thus, tone mapping based on non-linear mapping functions using global or local operators are preferable [1]. The log is a common non-linear remapping function, however, log-compressed images can have severe amplification of noise and have inaccurate results. To overcome this challenge, a global non-linear adjustment can be used,

$$\mathbf{I}_{rHDR} = Z_{max} \left(\frac{I_{\text{HDR}}}{(I_{\text{HDR}})_{max}}\right)^{\gamma} \tag{3}$$

where $Z_{\max} = 2^{12}$ to produce a 12-bit remapped HDR image, $\mathbf{I}_{\text{rHDR}}$.

In this work we use a log remapping function or a global linear adjustment with $0.5 < \gamma < 0.8$ depending on the dynamic range of the HDR image $\mathbf{I}_{\text{HDR}}$. Still sub regions of the HDR map can lack sufficient contrast and therefore details maybe hidden in visualization.

Histogram equalization is a transformation that is employed to flatten the pixel intensity distribution. Pixels' intensities are approximately uniformly distributed and the global contrast is enhanced. Because this method works in a global way, the remapped HDR image may still have low local contrast. To overcome this drawback, local operators, which offer higher detail preservation, were used.

Contrast Limited Adaptive Histogram Equalization (CLAHE) is a very effective implementation of adaptive histogram equalization algorithm [2] that exploits contrast limitation in order to enhance local contrast and reduces the negative effect of noise amplification. CLAHE implementation expects the image to be reduced into non-overlapping blocks for which individual histogram equalization transformation is applied. Blocks are then further processed through an interpolation to prevent artifacts on the edges of adjacent blocks. Moreover, in order to reduce noise artifacts, the histograms are clipped by user-defined parameters and values above threshold are redistributed evenly along the histogram. The two main parameters that are required by the CLAHE algorithm are the block size and the clipping level. Although automatic parameter selection methods exist (for instance based on entropy [2]), these parameters are commonly chosen manually as trade-off between noise amplification and contrast enhancement. Herein all remapped HDR images ($\mathbf{I}_{\text{rHDR}}$) are displayed by first applying the log or the global

operator in equation 3 on the original HDR image, followed, if necessary, by the local operator CLAHE algorithm.

Supplementary Note 3

Detector calibration. In this work we use a known sensor response based on measured calibration curves to reconstruct the HDR image rather than solving for the sensor response for each image, as is commonly done in photography or CCD based HDR [3]. Using a known calibration reduces computational time and improves reproducibility between all acquisitions. To measure the detector response a constant concentration of fluorescein was imaged with increasing input power at various gains. Two-photon and confocal calibration curves are shown in Supplementary Fig. 5.

Supplementary References

[1] Shan, Q., Jia, J. Y. & Brown, M. S. Globally optimized linear windowed tone mapping. *IEEE Trans. on Visualization and Computer Graphics*. **16**, 663-75 (2010).

[2] Min, B. S., Lim, D. K., Kim, S. J. & Lee J. Hj. A Novel Method of Determining Parameters of CLAHE Based on Image Entropy. *International Journal of Software Engineering and Its Applications* **7**, 113-120 (2013),

[3] Debevec, P. & Malik, J. Recovering high dynamic range radiance maps from photographs. In Proceedings of the 24th ACM annual conference on computer graphics and interactive techniques, Los Angeles, CA, USA 369-378 (1997).
